# Problem drinking, wellbeing and mortality risk in Chinese men: findings from the China Kadoorie Biobank

**DOI:** 10.1111/add.14873

**Published:** 2020-01-06

**Authors:** Pek Kei Im, Iona Y. Millwood, Yiping Chen, Yu Guo, Huaidong Du, Christiana Kartsonaki, Zheng Bian, Yunlong Tan, Jian Su, Yilei Li, Canqing Yu, Jun Lv, Liming Li, Ling Yang, Zhengming Chen, China Kadoorie Biobank (CKB) Collaborative Group

**Affiliations:** ^1^ Clinical Trial Service Unit and Epidemiological Studies Unit (CTSU), Nuffield Department of Population Health University of Oxford Oxford UK; ^2^ Medical Research Council Population Health Research Unit (MRC PHRU), Nuffield Department of Population Health University of Oxford Oxford UK; ^3^ Chinese Academy of Medical Sciences Beijing China; ^4^ Jiangsu CDC Jiangsu China; ^5^ Meilan CDC Hainan China; ^6^ Department of Epidemiology and Biostatistics, School of Public Health Peking University Beijing China

**Keywords:** Alcohol, anxiety, China, depression, mental health, mortality, problem drinking, sleep problems, stressful life events, wellbeing

## Abstract

**Aims:**

To assess the associations of problem drinking with wellbeing and mortality in Chinese men.

**Design:**

Population‐based prospective cohort study.

**Setting:**

Ten diverse areas across China.

**Participants:**

A total of 210 259 men aged 30–79 years enrolled into China Kadoorie Biobank between 2004 and 2008.

**Measurements:**

Self‐reported alcohol intake and indicators of problem drinking (i.e. drinking in the morning, unable to stop drinking, unable to work due to drinking, negative emotions after drinking, having shakes after stopping drinking) were assessed by questionnaire at baseline, along with stressful life events (e.g. divorce, income loss, violence) and wellbeing‐related measures (e.g. life satisfaction, sleep problems, depression, anxiety). Problem drinking was defined as reporting at least one of the drinking problem indicators. Follow‐up for mortality and hospitalized events was through linkage to death registries and national health insurance systems. Multivariate logistic regression models assessed cross‐sectional relationships between problem drinking and stressful life events/wellbeing. Cox proportional hazards regression models estimated prospective associations of problem drinking with mortality/hospitalized events.

**Findings:**

A third of men were current regular drinkers (i.e. drank alcohol at least weekly), 24% of whom reported problem drinking: 8% of all men. Experience of stressful life events in the past 2 years, especially income loss [odds ratio (OR) = 1.86, 95% confidence interval (CI) = 1.45–2.39], was associated with increased problem drinking. Compared with low‐risk drinkers (i.e. intake < 200 g/week, no reported problem drinking or habitual heavy drinking episodes), men with problem drinking had poorer self‐reported health, poorer life satisfaction and sleep problems, and were more likely to have symptoms of depression and anxiety. Men with two or more problem drinking indicators had an approximately twofold higher risk for all‐cause mortality as well as mortality and morbidity from external causes (i.e. injuries), respectively, and 15% higher risk for any hospitalization, compared with low‐risk drinkers (all *P* < 0.01).

**Conclusion:**

Eight per cent of men in China are problem drinkers, and this is associated with significantly increased risk of physical and mental health problems and premature death.

## Introduction

Alcohol drinking is a well‐recognized risk factor for the global burden of disease 1. Among regular heavy drinkers, a proportion may develop problem drinking, a maladaptive pattern of alcohol use causing problems such as impairment and distress, and eventually alcohol use disorder (AUD) 2, 3. Previous studies in western populations have shown that problem drinking, using definitions including heavy drinking, AUD and the experience of alcohol‐related problems, was associated with depression and anxiety 4, 5, 6, 7, 8, 9, sleep problems 10, 11, lower life satisfaction 12, 13, 14 and higher risk of mortality and suicide 15, 16. Also, it has been reported that stressful life events were associated with alcohol craving, binge drinking and problem drinking 17, 18, 19.

In China, the patterns of drinking differ importantly from western populations, including a lower prevalence of regular drinking especially among women, and the Chinese custom of drinking spirits and drinking alcohol with meals 20, 21, 22. The prevalence of alcohol dependence increased from 0.02 to 0.68% between the 1980s and 1990s 23, paralleled with a sharp increase in per‐capita alcohol consumption from 2.5 litres in 1978 to 7.2 litres in 2016 1, 24. In 2010, AUD was the ninth leading cause of disability, and the second most important mental disorder after depression in China 25. The relationships between problem drinking, wellbeing and health have emerged as important public health concerns, as comorbid AUD and mental disorders have been associated with higher risk of suicide attempts in western populations 26, 27. Despite this, large‐scale epidemiological evidence from Chinese populations is limited, with previous studies constrained by small sample size 28, crude measurement of alcohol drinking (i.e. user versus non‐user) 29, 30 and limited data on other health outcomes 31, 32.

Given the cultural differences in drinking patterns and the growing alcohol‐attributable disease burden, assessment of the relationships between problem drinking and health consequences in Chinese populations is needed to inform treatment and prevention strategies. This study uses data from the prospective China Kadoorie Biobank (CKB) of 0.5 million adults (210 259 men, 302 632 women) to (1) examine the prevalence and correlates of problem drinking; (2) assess the cross‐sectional relationships of problem drinking with experience of stressful life events and wellbeing; and (3) explore the prospective associations of problem drinking with all‐cause mortality, hospitalizations and events due to external causes. As few women (~2%) drink alcohol regularly in CKB 21, this study focused only on men.

## Methods

### Study design

Details of the CKB study design and methods have been previously reported 33, 34. Briefly, the study was conducted in 10 rural and urban regions among the general population in China, chosen for their diversity in exposure and disease patterns, while taking account of population stability and quality of death and disease registries. Overall, 512 891 adults aged 30–79 years were recruited during 2004–08 (response rate ~30%), among whom 210 259 (41%) were men and 56% were from rural areas. At local assessment clinics, trained health workers administered a laptop‐based questionnaire which included demographic and socio‐economic status, life‐style factors (smoking, drinking, diet, physical activity), stressful life events, wellbeing‐related measures and medical history; recorded physical measurements; and collected a blood sample. Ethical approval was obtained from local, national and international ethical committees. All participants provided written informed consent.

### Assessment of alcohol intake and problem drinking

Self‐reported alcohol drinking patterns and indicators of problem drinking at baseline were recorded by questionnaire, with details described previously 21, 35. In brief, participants were classified into: abstainers; ex‐regular drinkers; reduced‐intake drinkers; occasional drinkers; and current regular drinkers (i.e. drinking at least weekly in the past year). Current regular drinkers were asked further questions relating to their drinking patterns, e.g. frequency, amount consumed and experience of problem drinking indicators (see Supporting information, Table S1 for detailed definitions). Among current regular drinkers, problem drinking was defined by the report of one or more of the following indicators related to alcohol use in the past month: ever drinking in the morning; being unable to work or to do anything due to drinking; feeling depressed, irritated or losing control after drinking (i.e. negative emotions); being unable to keep away from drinking; and having shakes when stopping drinking. Heavy episodic drinking (HED) was defined as consuming more than 60 g of alcohol in a drinking session 1. Broadly following the US dietary guidelines 36 and based on the alcohol consumption during a typical drinking week, current regular drinkers who did not report any indicator(s) of problem drinking were categorized as: (1) low‐risk drinkers (drank alcohol < 200 g/week and no HED) or (2) high‐risk drinkers (drank alcohol ≥ 200 g/week or HED) (see Supporting information, Fig. S1 for flow‐chart of categorization of the study sample).

### Assessment of stressful life events and wellbeing

Experience of stressful life events in the past 2 years and wellbeing‐related measures (i.e. self‐reported health and life satisfaction at baseline, sleep problems, depression, anxiety) were assessed by baseline questionnaire. The 10 stressful life events (i.e. divorce, family conflict, death of spouse, death or major illness of a family member, job loss or retirement, bankruptcy, loss of income or debt, violence, major injury or traffic accident, natural disaster) are common stressors often examined in epidemiological research of stress and alcohol 18. Self‐reported poor health was defined by the report of self‐rated health as ‘poor’ (versus ‘fair’, ‘good’ or ‘excellent’). Life dissatisfaction was defined by the report of ‘being unsatisfied’ or ‘very unsatisfied’ with life (versus ‘neither satisfied nor dissatisfied’, ‘satisfied’ or ‘very satisfied’). Comparable to the standardized criteria of insomnia used in International Classification of Sleep Disorders (ICSD) 37, sleep problems were defined by the report of any of the following symptoms during the last month: difficulty in initiating or maintaining sleep; early‐morning awakenings; difficulty in staying alert during daytime; use of sleep aid medications. Major depressive episode (MDE) and general anxiety disorder (GAD) were assessed using the modified Chinese version of the World Health Organization 12‐month Composite International Diagnostic Interview‐Short Form (CIDI‐SF), triggered by positive responses to screening questions 38. For MDE, participants were asked if they had experienced any of the following depression symptoms for more than 2 weeks in the past year: feeling sad or depressed; loss of interest; loss of appetite; and feeling worthless. Participants reporting at least one symptom were further assessed for MDE using the CIDI‐SF (A). For GAD, participants who reported continuous anxiety lasting at least 1 month in the past year triggered further assessment for GAD by the CIDI‐SF (B). Those who responded positively to the screening questions but did not meet the CIDI‐SF diagnostic criteria of MDE or GAD were classified as having ‘depressive symptoms’ or ‘anxiety symptom’, respectively. Symptoms of panic attack and phobia in the past year were also recorded (see detailed questionnaire at http://www.ckbiobank.org).

### Mortality and morbidity follow‐up

Cause‐specific mortality was monitored through China's Centre for Disease Control (CDC) Disease Surveillance Points (DSP) system, with annual active confirmation through local residential, medical, health insurance and administrative records. Non‐fatal outcomes of major diseases and any episodes of hospitalization were collected through linkage with disease registries and the Chinese National Health Insurance claim databases. All events were coded using International Classification of Diseases (ICD)‐10 by trained staff blinded to baseline information. The follow‐up outcomes in this study were all‐cause mortality, any episode of hospitalization and all fatal and non‐fatal events due to all external causes (ICD‐10: V01‐Y98). By 1 January 2016, 21 545 (10.3% of all men) men had died and 1792 (0.9%) were lost to follow‐up.

### Statistical analysis

Means and percentages of baseline characteristics were adjusted for age and region where appropriate by direct standardization to the age and region structure of the cohort. The statistical associations between problem drinking categories and baseline characteristics were tested using multinomial logistic regression for categorical variables, logistic regression for binary variables and linear regression for continuous variables. Logistic regression was used to calculate odds ratios (ORs) of problem drinking associated with exposure to stressful life events, and ORs of wellbeing‐related measures associated with exposure to problem drinking and individual problem drinking indicators, in cross‐sectional analyses among current regular drinkers adjusted for age group, region, education, income and smoking. For analyses of problem drinking and wellbeing‐related measures, marital status, prior chronic diseases and body mass index (BMI) were additionally adjusted for (see Supporting information, Table S2 for details of analytical models).

Analyses of the prospective associations between problem drinking and health outcomes were restricted to current regular drinkers without major chronic diseases at baseline (*n* = 57 166) (Supporting information, Table S2). Cox proportional hazards regression, stratified by age‐at‐risk (5‐year intervals) and 10 regions, and adjusted for education, income, smoking, fruit intake, physical activity and BMI, was used to estimate hazard ratios (HRs) for all‐cause mortality, any hospitalization and events due to external causes associated with problem drinking and individual problem drinking indicators. The proportional hazards assumption for the Cox model was checked using scaled Schoenfeld residuals and by examining the HRs for the first 4 years and for subsequent years of follow‐up 39.

For analyses involving more than two levels of exposure, floating standard errors were used to estimate group‐specific variances and confidence intervals (CI) of log ORs or log HRs for all categories including the reference group, which reflected independent variability within each group. It enabled comparison between any two categories rather than simply pairwise comparisons with the reference category 40, 41. Heterogeneity across problem drinking categories was assessed with a χ^2^ test, with the null hypothesis that all coefficients tested are the same.

Sensitivity analyses were conducted to explore the associations of problem drinking with depression and anxiety after removing the negative emotions indicator from the definition of problem drinking. Analyses of the associations of problem drinking with stressful life events, wellbeing and mortality risk with further adjustment for potential confounding factors, e.g. prior chronic diseases for stressful life events, physical activity for wellbeing and prior psychiatric disorders for all analyses, were performed. Further analyses on the associations of problem drinking with wellbeing and mortality risk were repeated among all male participants.

SAS version 9.4 was used for performing statistical analyses and R version 3.4.0 was used for producing figures. The analyses were not pre‐registered and the results should be considered exploratory.

## Results

Of the 210 259 men recruited at baseline, 33% (*n* = 69 904) drank alcohol regularly, among whom the mean age was 51 years and 50% were from urban areas (Table 1). Among current regular drinkers, 24% reported at least one indicator of problem drinking (8% of all men), i.e. problem drinkers. Compared with low‐risk drinkers, problem drinkers tended to have less education and lower household income (*P* < 0.001). Problem drinkers reported the following indicators: morning drinking (55%) and unable to stop drinking (47%), which increased with age (*P* < 0.001), unable to work due to drinking (15%), negative emotions after drinking (7%) and having shakes when stopping drinking (2%), which decreased with age (*P* < 0.001) (Supporting information, Fig. S2). Among all men, the prevalence of problem drinking varied from 2% in Suzhou to 19% in Sichuan (Supporting information, Fig. S3), while among current regular drinkers it varied from 5% in Suzhou to 37% in Sichuan (Supporting information, Fig. S4). Overall, problem drinking was generally more common in rural than urban areas (10 versus 5% among all men, 34 versus 14% among current regular drinkers). This was driven mainly by the higher prevalence of morning drinking in rural areas (61 versus 39% among problem drinkers) (Supporting information, Fig. S5).

**Table 1:** Baseline characteristics of male current regular drinkers by problem drinking status.

		Non‐problem drinkers		Problem drinkers		
	All current regular drinkers	Low‐risk drinkers (Within‐guidelines)	High‐risk drinkers	1 indicator	2+ indicators	*P*‐value
Number of men (%)	69 904	25 958 (37.1)	27 254 (39.0)	13 271 (19.0)	3421 (4.9)	
Socio‐demographic characteristics						
Mean age, years (SD)	51.1 (10.2)	51.7 (10.7)	50.2 (9.8)	51.8 (10.1)	50.9 (9.8)	< 0.001
Age groups, years, %						< 0.001
< 40	14.1	14.7	15.4	12.4	14.7	
40–49	32.4	31.2	35.2	31.2	33.2	
50–59	31.6	29.8	31.4	33.0	34.6	
60–69	16.3	17.6	14.0	17.6	14.2	
70+	5.5	6.8	4.0	5.7	3.2	
Urban area, %	50.1	61.3	51.9	30.5	24.0	< 0.001
Highest education, %						< 0.001
Primary or below	39.7	36.9	40.0	42.3	42.5	
Middle or high school	52.2	53.6	51.9	51.8	53.4	
Technical school, college or university	8.1	9.5	8.1	5.9	4.1	
Household income, yuan/year, %						< 0.001
< 20 000	48.5	47.3	47.2	52.5	53.2	
20 000–34 999	28.0	28.0	28.1	26.8	28.1	
35 000+	23.5	24.6	24.7	20.6	18.7	
Married, %	93.8	94.8	93.9	92.6	91.1	< 0.001
Life‐style and physical measurements						
Regular smoking, %	71.3	64.9	74.1	76.2	79.0	< 0.001
Mean physical activity, MET‐h/d^a^ (SD)	22.9 (15.0)	22.9 (14.8)	22.8 (14.9)	23.1 (15.2)	23.4 (15.7)	0.028
Mean SBP, mmHg (SD)	134.2 (19.8)	131.8 (19.1)	135.7 (19.7)	135.3 (20.6)	136.7 (20.9)	< 0.001
Mean BMI, kg/m^2^ (SD)	23.7 (3.2)	23.6 (3.2)	23.9 (3.3)	23.5 (3.2)	23.3 (3.1)	< 0.001
Medical history, %						
CHD	1.9	2.0	1.7	1.9	3.0	0.012
Stroke or TIA	1.3	1.3	1.2	1.4	2.4	0.058
Cancer	0.2	0.3	0.2	0.2	0.2	0.550
Chronic hepatitis or cirrhosis	1.2	1.2	1.1	1.4	1.4	0.013
Diabetes	2.0	2.1	2.0	1.8	2.5	0.155
Psychiatric disorder	0.2	0.1	0.1	0.2	0.4	0.481

SD = standard deviation; MET‐h/d = metabolic equivalent of task per hour per day; SBP = systolic blood pressure; DBP = diastolic blood pressure; BMI = body mass index; CHD = coronary heart disease; TIA = transient ischaemic attack; HED = heavy episodic drinking.

Prevalences and means are adjusted for age group and region as appropriate.

*P*‐values were calculated using a χ^2^ test for association between problem drinking and baseline characteristics.

Low‐risk drinkers were current regular drinkers who drank < 200 g/week, with no HED in a typical drinking week or problem drinking indicator reported; high‐risk drinkers were current regular drinkers who either drank at least 200 g/week or engaged in HED in a typical drinking week, but with no problem drinking indicator reported; problem drinkers were current regular drinkers who reported at least one problem drinking indicator, and were further classified into ‘1 problem drinking indicator’ and ‘2+ problem drinking indicators’ according to the number of problem drinking indicators reported.

a The sum of MET‐h/d was estimated based on questions on the usual type and duration of activities related to work, commuting, household chores and leisure‐time exercise in the past year, adapted from validated questionnaires used in previous cohort studies, with some additional modifications after a China Kadoorie Biobank (CKB) pilot study.

Compared with low‐risk drinkers, problem drinkers consumed on average more than three times as much alcohol each week (> 383 versus 109 g/week) and were more likely to drink daily (*P* < 0.001) (Table 2). High‐risk drinkers had a similar mean weekly consumption to problem drinkers with one indicator. Among problem drinkers, those reporting two or more indicators had higher mean alcohol intake and higher frequency of daily drinking and HED. This was seen in both rural and urban areas, although the overall mean alcohol consumption level and prevalence of HED in a typical drinking week were higher in rural than urban areas (Supporting information, Table S3). The highest mean consumption was observed in problem drinkers reporting having shakes when stopping drinking, followed by morning drinking and negative emotions after drinking (Supporting information, Table S4). Those reporting negative emotions or having shakes were most likely to report more than one drinking problem (Supporting information, Table S4).

**Table 2:** Drinking characteristics by problem drinking status in male current regular drinkers.

	Low‐risk drinkers (*n* = 25 958)			High‐risk drinkers (*n* = 27 254)		Problem drinkers with 1 indicator (*n* = 13 271)	Problem drinkers with 2+ indicators (*n* = 3421)			*P*‐value
Drinking characteristics										
Drank alcohol on the day of survey, %	10.7			17.5		28.5		33.7		< 0.001
Daily drinking, %	35.9			74.5		76.8		83.7		< 0.001
Age started regular drinking, mean (SD)	30.8 (12.2)			27.5 (9.5)		27.5 (10.7)		26.1 (9.4)		< 0.001
Drinking with meals, %	87.0			86.4		85.0		83.5		< 0.001
Flushing response^a^, %	25.5			13.8		15.6		13.8		< 0.001
Typical drinking week										
Mean weekly consumption, g/week (SD)	109.2 (56.5)			378.4 (209.1)		383.4 (277.6)		506.5 (344.4)		< 0.001
Heavy episodic drinking (HED), %	0.0			60.8		48.4		61.0		< 0.001
Heavy drinking (200 + g/week), %	0.0			88.0		69.8		80.7		< 0.001
HED and heavy drinking, %	0.0			48.8		45.3		58.4		< 0.001
Beverage types consumed, %										< 0.001
Strong spirit (≥ 40% alcohol) only	34.8			56.4		51.8		55.7		
Weak spirit (< 40% alcohol) only	21.1			24.1		24.0		23.8		
Beer only	27.2			12.3		12.6		10.6		
Rice wine or grape wine only	16.8			7.1		11.5		9.9		
Special occasion										
Mean consumption per session, g/session (SD)	106.4 (86.3)			169.2 (111.0)		168.3 (112.0)		203.5 (134.4)		< 0.001
HED on special drinking occasion, %	69.4			93.5		89.2		94.2		< 0.001
Last time drinking										
Mean consumption per session, g/session (SD)	32.0 (26.7)			61.0 (41.8)		57.6 (40.1)		67.0 (47.5)		< 0.001
HED on the last drinking day, %	11.4			41.3		38.4		46.0		< 0.001

SD = standard deviation; HED = heavy episodic drinking.

Prevalences and means are adjusted for age group and region.

*P*‐values were calculated using a χ^2^ test for association between problem drinking and drinking characteristics.

Low‐risk drinkers were current regular drinkers who drank < 200 g/week, with no HED in a typical drinking week or problem drinking indicator reported; high‐risk drinkers were current regular drinkers who either drank at least 200 g/week or engaged in HED in a typical drinking week, but with no problem drinking indicator reported; problem drinkers were current regular drinkers who reported at least one problem drinking indicator, and were further classified into ‘1 problem drinking indicator’ and ‘2+ problem drinking indicators’ according to the number of problem drinking indicators reported.

a Experiencing hot flushes or dizziness soon after first mouthful or after drinking small amount of alcohol.

Experience of any stressful life events was associated with an adjusted OR of 1.24 (95% CI = 1.16–1.33) for problem drinking (Table 3), compared with those who had not experienced stressful life events. Of the 10 stressful life events surveyed, loss of income or debt (OR = 1.86, 95% CI = 1.45–2.39) and experience of violence (OR = 1.68, 95% CI = 1.11–2.56) had the strongest associations with problem drinking. Family conflict, death or major illness of a family member, job loss or retirement and bankruptcy were also associated with problem drinking. Divorce was associated with problem drinking among urban but not rural drinkers, while the associations between problem drinking with finance‐related events were significant among rural drinkers only (Supporting information, Table S5).

**Table 3:** Cross‐sectional associations of stressful life events with problem drinking in male current regular drinkers.

	Total regular drinkers *n*	Problem drinkers^a^ *n* (%)	Adjusted OR (95% CI) for problem drinking	*P‐*value
**Family‐related events**				
Divorce or separation				
No	69 701	16 639 (23.9%)	1.00	
Yes	203	53 (26.1%)	1.32 (0.95–1.83)	0.1027
Family conflict				
No	69 417	16 540 (23.8%)	1.00	
Yes	487	152 (31.2%)	1.50 (1.22–1.84)	0.0001
Death of spouse				
No	69 550	16 596 (23.8%)	1.00	
Yes	354	96 (27.1%)	1.07 (0.83–1.36)	0.6133
Death or major illness of other family member				
No	66 680	15 871 (23.8%)	1.00	
Yes	3224	821 (25.5%)	1.20 (1.10–1.30)	< 0.0001
Any family‐related events				
No	65 748	15 606 (23.7%)	1.00	
Yes	4156	1086 (26.1%)	1.21 (1.13–1.31)	< 0.0001
**Finance‐related events**				
Job loss or retirement				
No	69 587	16 622 (23.9%)	1.00	
Yes	317	70 (22.1%)	1.45 (1.09–1.91)	0.0102
Bankruptcy				
No	69 683	16 608 (23.8%)	1.00	
Yes	221	84 (38.0%)	1.48 (1.11–1.96)	0.0068
Loss of income or debt				
No	69 592	16 577 (23.8%)	1.00	
Yes	312	115 (36.9%)	1.86 (1.45–2.39)	< 0.0001
Any finance‐related events				
No	69 099	16 441 (23.8%)	1.00	
Yes	805	251 (31.2%)	1.57 (1.34–1.84)	< 0.0001
**Injury‐related events**				
Violence				
No	69 796	16 653 (23.9%)	1.00	
Yes	108	39 (36.1%)	1.68 (1.11–2.56)	0.0149
Major injury or traffic accident				
No	69 403	16 566 (23.9%)	1.00	
Yes	501	126 (25.1%)	1.15 (0.93–1.43)	0.1929
Natural disaster				
No	69 848	16 679 (23.9%)	1.00	
Yes	56	13 (23.2%)	1.05 (0.54–2.02)	0.8869
Any injury or disaster events				
No	69 244	16 516 (23.9%)	1.00	
Yes	660	176 (26.7%)	1.22 (1.01–1.47)	0.0347
**Any major stressful life event**				
No	64 509	15 266 (23.7%)	1.00	
Yes	5395	1426 (26.4%)	1.24 (1.16–1.33)	< 0.0001

OR = odds ratio; CI = confidence interval.

ORs were adjusted for age group, region, education, income and smoking status. All *P*‐values are from a Wald χ^2^ test.

a Reporting one or more in the past month of: drinking in the morning; unable to work or do anything due to drinking; depressed, irritated or lost control due to drinking; could not stop drinking; had shakes when stopping drinking.

**Figure 1:**
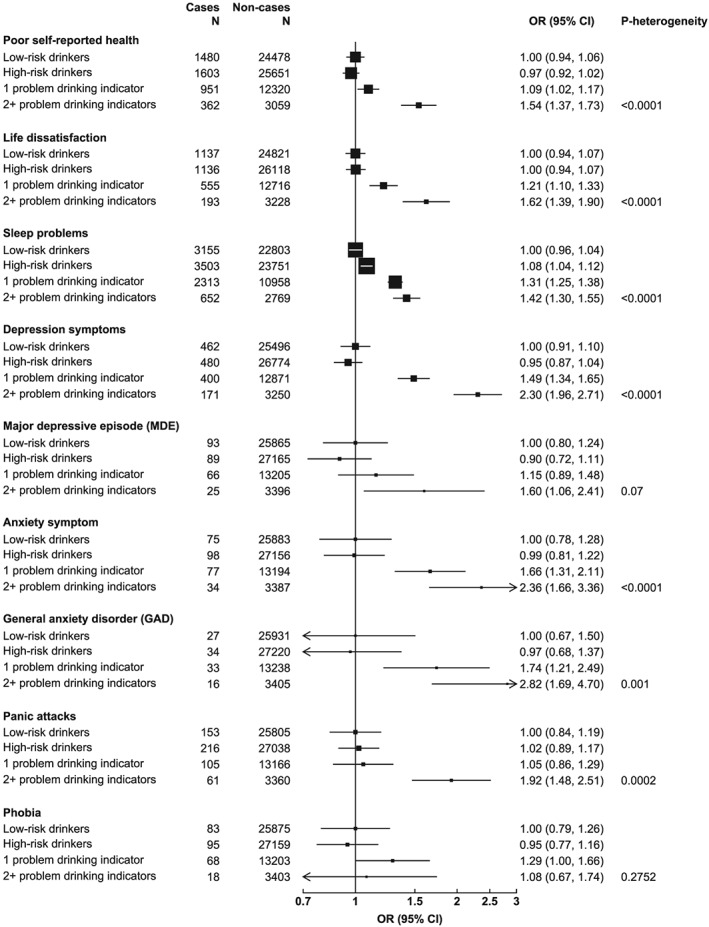
Cross‐sectional associations of problem drinking with wellbeing‐related measures in 69 904 male current regular drinkers. Odds ratios (ORs) were adjusted for age group, region, education, income, marital status, prior chronic diseases, smoking and body mass index (BMI). Each solid square represents an OR; 95% confidence intervals (CIs) are plotted using floating standard errors to allow for comparison between any two categories. The size of each box is inversely proportional to the ‘floated’ variance of the log OR in each group and the error bars indicate the group‐specific 95% CI. HED = heavy episodic drinking. Low‐risk drinkers were current regular drinkers who drank < 200 g/week, with no HED in a typical drinking week or problem drinking indicator reported; high‐risk drinkers were current regular drinkers who either drank at least 200 g/week or engaged in HED in a typical drinking week, but with no problem drinking indicator reported; problem drinkers were current regular drinkers who reported at least one problem drinking indicator, and were further classified into ‘1 problem drinking indicator’ and ‘2+ problem drinking indicators’ according to the number of problem drinking indicators reported.

There were positive associations between problem drinking and poor wellbeing (Fig. 1), with apparently stronger associations among urban than rural drinkers (Supporting information, Fig. S6). Compared with low‐risk drinkers, those with two or more indicators had approximately 1.5‐fold higher risks for poor self‐reported health, life dissatisfaction and sleep problems (all *P* < 0.001). The associations were stronger for other wellbeing‐related measures, i.e. an approximately 2–2.4‐fold higher risk for panic attacks, symptoms of depression and anxiety (all *P* < 0.001). The associations of problem drinking with MDE and GAD were similar to those with symptoms of depression and anxiety respectively, although the ORs were less extreme for MDE. Among problem drinkers, there were significant dose–response relationships of poor wellbeing with the number of indicators reported (*P* < 0.05 for all except GAD and phobia, Supporting information, Table S6). High‐risk drinking was not associated with most wellbeing‐related measures when compared to low‐risk drinking, except for a higher likelihood of early‐morning awakening (Supporting information, Table S7). Each individual problem drinking indicator was associated with poor wellbeing when compared with low‐risk drinking. In particular, negative emotions after drinking and having shakes when stopping drinking were each associated with a more than threefold higher risk of depression symptoms (Supporting information, Table S8). After removing the negative emotions indicator from the definition of problem drinking, the associations between problem drinking and symptoms of depression and anxiety were attenuated, but remained significant (Supporting information, Fig. S7).

**Figure 2:**
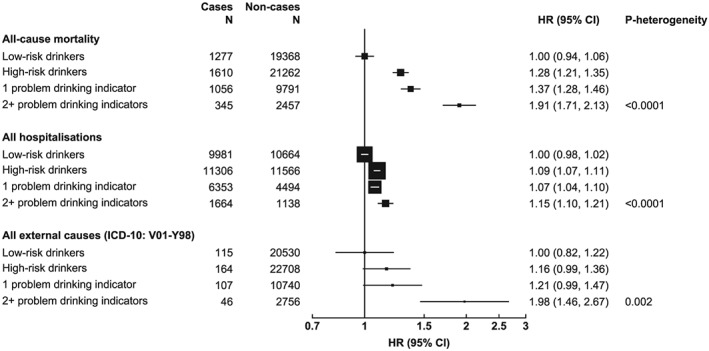
Prospective associations of problem drinking with all‐cause mortality, all hospitalizations and events due to all external causes in 57 166 male current regular drinkers without prior chronic diseases. Models were stratified by age‐at‐risk and region, further adjusted for education, income, smoking, physical activity, fruit intake and body mass index (BMI). Participants with prior coronary heart disease, stroke, transient ischaemic attack, diabetes, cancer, tuberculosis, chronic hepatitis/cirrhosis, rheumatoid arthritis, peptic ulcer, emphysema/bronchitis, gallstone/gallbladder disease or kidney disease were excluded from the analysis. Each solid square represents a hazard ratio (HR); 95% confidence intervals (CIs) are plotted using floating standard errors to allow for comparison between any two categories. The size of each box is inversely proportional to the ‘floated’ variance of the log HR in each group and the error bars indicate the group‐specific 95% CI. ICD‐10 = International Classification of Diseases version 10; HED = heavy episodic drinking. Low‐risk drinkers were current regular drinkers who drank < 200 g/week, with no HED in a typical drinking week or problem drinking indicator reported; high‐risk drinkers were current regular drinkers who either drank at least 200 g/week or engaged in HED in a typical drinking week, but with no problem drinking indicator reported; problem drinkers were current regular drinkers who reported at least one problem drinking indicator, and were further classified into ‘1 problem drinking indicator’ and ‘2+ problem drinking indicators’ according to the number of problem drinking indicators reported.

The risk of all‐cause mortality was associated with problem drinking, with adjusted HRs of 1.00 (95% CI = 0.94–1.06) for low‐risk drinkers, 1.28 (95% CI = 1.21–1.35) for high‐risk drinkers, 1.37 (95% CI = 1.28–1.46) and 1.91 (95% CI = 1.71–2.13) for drinkers with one and two or more problem drinking indicators, respectively (Fig. 2). The patterns of associations of problem drinking with incident events due to external causes were broadly similar to those with all‐cause mortality. There were also slightly increased risks for overall hospitalizations among high‐risk and problem drinkers, with higher HRs in urban than rural areas (Supporting information, Fig. S8). The risk of mortality and hospitalizations increased progressively with the number of problem drinking indicators reported (*P* < 0.01, Supporting information, Table S9). For individual problem drinking indicators, having shakes when stopping drinking was associated with the highest HR of all‐cause mortality, followed by morning drinking and negative emotions after drinking (Supporting information, Table S10).

The main results from the cross‐sectional and prospective analyses persisted after further adjustment for other relevant covariates such as prior chronic diseases, physical activity and prior psychiatric disorders (Supporting information, Tables S11–S13). Repeating the analyses in all men showed a higher risk of poor wellbeing measures and mortality in abstainers and ex‐regular drinkers, who were older and had a higher prevalence of prior chronic diseases and psychiatric disorders at baseline (Supporting information, Table S14), compared with low‐risk drinkers (Figs S9–S10).

## Discussion

In this large study of Chinese adults, one in four men who drank alcohol regularly experienced at least one indicator of problem drinking. Problem drinking was more prevalent among men living in rural areas and with lower socio‐economic status. Experience of stressful life events, especially loss of income, was associated with a higher risk of problem drinking. Problem drinking was associated with poor wellbeing and higher risk of all‐cause mortality and incident events due to external causes.

The extremely low prevalence of regular drinking in women (~2%) in CKB suggested that the burden of harmful alcohol use is likely to be highly skewed towards Chinese men. The overall prevalence of problem drinking in men (8%) in this study is broadly consistent with a previous meta‐analysis of 38 cross‐sectional studies, most of which focused on a single geographical region, including 1 304 354 individuals conducted during 1987–2013 in China, which reported a prevalence of AUD of 10% in men and 0.1% in women 42. Furthermore, we found that the prevalence of problem drinking varied greatly among geographic regions, with a generally higher prevalence in rural than urban regions, as shown in the previous meta‐analysis in China (overall AUD prevalence: 6.1 in rural versus 5.2% in urban) 42. The rural–urban divide in our study may have been due to the heavier alcohol consumption in rural than urban drinkers 21, and was largely driven by the high frequency of morning drinkers in rural areas. In addition, we found that negative emotions and being unable to work after drinking were more prevalent in urban and younger men, among whom we previously reported an increase in the prevalence of problem drinking between 2004–08 and 2013–14 35, reflecting the different contexts of problem drinking across regions and generations in China. In line with studies in western countries 8, 9, we found that problem drinking prevalence tended to be higher among those with lower socio‐economic status, while previous evidence from China was inconsistent and the associations varied greatly throughout different regions 28, 43, 44, 45.

Previous studies of mainly western populations have reported the associations of stressful life events, particularly financial‐related events, e.g. job loss, with alcohol craving and problem drinking 18, 19, 46, 47. Two studies on the changes in alcohol use patterns during the 2008 economic crisis in European Union countries 48 and in a US sample of 2 million adults 49 showed that the economic crisis was linked with increased rates of alcohol misuse and alcohol‐related mortality and hospitalization, particularly among the unemployed. Our study provides clear evidence that the experience of stressful life events over the past 2 years, particularly financial stress, was associated with an increased likelihood of past‐month problem drinking in Chinese men, although these findings were cross‐sectional and the directionality of the associations is still unknown. The regional differences we observed suggested that there may be contextual factors influencing the relationships between stress and problem drinking, e.g. lack of financial support in the more deprived, rural drinker groups.

Although the evidence is limited in China, previous studies which are mainly from western populations have shown associations of problem drinking with various measures of poor wellbeing, including lower life satisfaction, sleep problems, depression and anxiety 4, 7, 11, 12, 13, 14, 50, 51, 52. A previous study of 16 255 adults aged > 65 years in China found that low life satisfaction was related to higher likelihood of alcohol drinking, but without further investigation into consumption level or problem drinking 30. In our study, we demonstrated that, among Chinese men, drinking problems were associated with life dissatisfaction and also other measures of poor wellbeing. We found no association between high‐risk drinking and poor wellbeing. This was in line with a recent Dutch study of 6705 adults, in whom lower life satisfaction was associated with problem drinking but not the alcohol amount consumed 53. Therefore, our results suggest that the relationships between problem drinking and most wellbeing‐related measures may not be due solely to the amount of alcohol consumed *per se*, but to the negative consequences of, or the genetic predisposition to, problem drinking 53, 54. One exception may be sleep problems, for which we showed that high‐risk drinkers had an increased likelihood of early‐morning awakening. This was in line with clinical studies, which suggested that the quantity of alcohol consumption may have a direct physiological role on underlying sleep quality 11.

The associations between problem drinking and depression and anxiety disorders have been established previously among western populations, in both cross‐sectional and prospective studies 4, 5, 6, 7, 8, 9, 55, 56, while in China the evidence is still limited. A recent study of 74 752 Chinese men combining surveys from five provinces reported an inverse association between current AUD and mood disorders (OR = 0.6, 95% CI = 0.4–0.8) and anxiety disorders (OR = 0.5, 95% CI = 0.3–1.0) in men, with limited adjustment for age only 31. In contrast, our study covering 10 diverse regions provided strong evidence of the associations between problem drinking and higher risk of depression and anxiety among male regular drinkers. Apart from differences in study regions and covariate adjustment, the contrasting results between the two Chinese studies may be explained by methodological issues. First, the exclusion of substance‐induced mental disorders in the previous study might have attenuated the cross‐sectional relationships between problem drinking and mental disorders. Furthermore, unlike the previous study in China, we have excluded non‐drinkers, some of whom may have abstained from alcohol because of old age or health reasons and thus may have poorer wellbeing and higher risk of disease outcomes due to reverse causality, as suggested in our sensitivity analyses. Another study of 15 628 Chinese participants reported an inverse association between baseline alcohol drinking and subsequent depression symptoms (OR = 0.6, 95% CI = 0.5–0.7) 32. However, further investigation among drinkers suggested an increased risk of persistent depressive symptoms associated with alcohol withdrawal (OR = 2.2, 95% CI = 0.9–5.5). This suggests that the relationships between problem drinking and depression might only become apparent when investigated among regular drinkers, where potential reverse causality by sick non‐starters is avoided. Our findings suggested that the associations between problem drinking and poor wellbeing might be more pronounced among urban drinkers, which may be related to the more serious types of drinking problem (e.g. unable to work) experienced among urban problem drinkers. In addition, we showed that negative emotions after drinking and having shakes when stopping drinking were most strongly associated with poor wellbeing, which may be explained by the higher severity of problem drinking (i.e. number of other problem drinking indicators) involved.

For long‐term health outcomes, our findings were generally consistent with previous studies world‐wide 15, 16, 57, 58, 59, including a meta‐analysis which reported a risk ratio (RR) for all‐cause mortality of 1.91 (95% CI = 1.51–2.42), comparing men with and without AUD 15, and another meta‐analysis reporting a RR of 1.74 (95% CI = 1.26–2.21) for completed suicides 16. As well as having shakes when stopping drinking and negative emotions after drinking, we found that morning drinking was also strongly associated with all‐cause mortality, which was due probably to the high alcohol consumption level among morning drinkers. In this study, the excess risk of all‐cause hospitalizations associated with problem drinking was modest, possibly diluted by non‐alcohol‐related conditions.

Several possible mechanisms have been proposed to explain the pathway linking problem drinking and poor mental health, including the self‐medication theory (i.e. mental illness causes problem drinking); substance‐induced pathway (i.e. problem drinking causes mental illness via neurophysiological and/or psychosocial impacts); a reciprocal causal relationship (i.e. problem drinking and mental illness increases the risk of the other simultaneously); or causation by a common factor (i.e. the co‐occurrence of problem drinking and mental illness is explained by a third factor, e.g. genetics or environment) 4, 55. For health outcomes, the excess overall mortality risk may arise from the direct effects of alcohol misuse (e.g. high levels of intake, intoxication) and possible genetic predisposition to both problem drinking and adverse health outcomes (e.g. impulsive personality) 58.

The chief strengths of this study were the large sample size, the wide range of geographical areas covered and detailed data collected on drinking patterns and wellbeing‐related measures. However, the study has several limitations. First, CKB was not designed to be nationally representative and, given the overall response rate of ~30%, the present study may have underestimated the prevalence of problem drinking, as individuals with severe problem drinking may have been less likely to participate in the CKB. However, the associations of problem drinking with stressful life events, wellbeing and health outcomes are likely to be generalizable to the general population, given the large size and diversity of CKB. Secondly, the low prevalence of regular drinking among women precluded reliable exploration of associations in women. Thirdly, full clinical diagnostic standard assessment for AUD (i.e. DSM‐5) was not used. Nevertheless, the construct of alcohol‐related problems used in our assessment was comparable to the major domains of AUD diagnostic criteria (Supporting information, Table S15). Furthermore, the heavy drinking patterns of problem drinkers and the similar prevalence of problem drinking reported here, as in a previous meta‐analysis on AUD prevalence in China 42, suggested a high‐quality problem drinking assessment. Fourthly, the temporal order and causal relationship between problem drinking, stressful life events and wellbeing could not be established using cross‐sectional analyses, and detailed exploration of this is beyond the scope of this study. Lastly, although careful adjustment was made for potential confounders, uncontrolled residual confounding (e.g. parental history of alcohol misuse) might remain.

In summary, this study showed that problem drinking was common among Chinese men, especially those with lower socio‐economic status. Problem drinking was associated with stressful life events, poor wellbeing and excess risks of overall mortality and accidents, injury and violence. Policy and public health actions are needed to tackle the issue of problem drinking in China, especially among men, to reduce the burden of harmful consequences.

## Declaration of interests

None.

## Supporting information


**Figure S1** Alcohol drinking status and problem drinking categorisation of study sample among CKB men.
**Figure S2.** Prevalence of problem drinking among male current regular drinkers, and of specific problem drinking indicators among problem drinkers, by age group.
**Figure S3.** Prevalence of current regular drinking and problem drinking among all men.
**Figure S4.** Prevalence of problem drinking among male current regular drinkers, and of specific problem drinking indicators among problem drinkers, by ten study regions.
**Figure S5.** Prevalence of problem drinking among male current regular drinkers, and of specific problem drinking indicators among problem drinkers, by rural and urban areas.
**Figure S6.** Cross‐sectional associations of problem drinking with wellbeing in male current regular drinkers, by rural and urban regions.
**Figure S7.** Cross‐sectional associations of problem drinking (excluding negative emotions indicator from the definition of problem drinking) with wellbeing in male current regular drinkers.
**Figure S8.** Prospective associations of problem drinking with all‐cause mortality, all hospitalisations and events due to all external causes in male current regular drinkers without prior chronic diseases, by rural and urban regions.
**Figure S9.** Cross‐sectional associations of problem drinking with wellbeing‐related measures in men.
**Figure S10.** Prospective associations of problem drinking with all‐cause mortality, all hospitalisations and events due to all external causes in men without prior chronic diseases.
**Table S1** Definitions of main alcohol drinking categories, pattern and problem drinking.
**Table S2** Details of main analytic models.
**Table S3** Drinking characteristics by problem drinking status in male current regular drinkers, by rural–urban regions.
**Table S4** Drinking characteristics by specific problem drinking indicators in male current regular drinkers.
**Table S5** Cross‐sectional associations of stressful life events with problem drinking in male current regular drinkers, by rural and urban regions.
**Table S6** Adjusted odds ratios (ORs) associated with an increase in the number of problem drinking indicators in men with at least one problem drinking indicator.
**Table S7** Cross‐sectional associations of problem drinking with sleep problems in male current regular drinkers.
**Table S8** Cross‐sectional associations of problem drinking indicators with wellbeing‐related measures in male current regular drinkers.
**Table S9** Adjusted hazard ratios (HRs) associated with per an increase in the number of problem drinking indicators in men with at least one problem drinking indicator.
**Table S10** Prospective associations of problem drinking indicators with all‐cause mortality, all hospitalisations, and events due to external causes in male current regular drinkers.
**Table S11** Cross‐sectional associations of stressful life events with problem drinking in male current regular drinkers in sequentially adjusted models.
**Table S12** Cross‐sectional associations of problem drinking with wellbeing‐related measures in male current regular drinkers in sequentially adjusted models.
**Table S13** Prospective associations of problem drinking with all‐cause mortality, all hospitalisations, and events due to external causes in male current regular drinkers in sequentially adjusted models.
**Table S14** Baseline characteristics of men by alcohol drinking and problem drinking status.
**Table S15** Comparison of problem drinking and AUD definitions in the China Kadoorie Biobank and commonly used screening tests and diagnostic criteria.
