# Supplementary material for: Problem drinking, wellbeing and mortality risk in Chinese men: findings from the China Kadoorie Biobank
Source: Addiction. 2020 Jan 6;115(5):850–62. doi: 10.1111/add.14873 (PMC7156287; doi:10.1111/add.14873)
Supplement: Supplementary file 1 — Figure S1 Alcohol drinking status and problem drinking categorisation of study sample among CKB men. Figure S2. Prevalence of problem drinking among male current regular drinkers, and of specific problem drinking indicators among problem drinkers, by age group. Figure S3. Prevalence of current regular drinking and problem drinking among all men. Figure S4. Prevalence of problem drinking among male current regular drinkers, and of specific problem drinking indicators among problem drinkers, by ten study regions. Figure S5. Prevalence of problem drinking among male current regular drinkers, and of specific problem drinking indicators among problem drinkers, by rural and urban areas. Figure S6. Cross‐sectional associations of problem drinking with wellbeing in male current regular drinkers, by rural and urban regions. Figure S7. Cross‐sectional associations of problem drinking (excluding negative emotions indicator from the definition of problem drinking) with wellbeing in male current regular drinkers. Figure S8. Prospective associations of problem drinking with all‐cause mortality, all hospitalisations and events due to all external causes in male current regular drinkers without prior chronic diseases, by rural and urban regions. Figure S9. Cross‐sectional associations of problem drinking with wellbeing‐related measures in men. Figure S10. Prospective associations of problem drinking with all‐cause mortality, all hospitalisations and events due to all external causes in men without prior chronic diseases. Table S1 Definitions of main alcohol drinking categories, pattern and problem drinking. Table S2 Details of main analytic models. Table S3 Drinking characteristics by problem drinking status in male current regular drinkers, by rural–urban regions. Table S4 Drinking characteristics by specific problem drinking indicators in male current regular drinkers. Table S5 Cross‐sectional associations of stressful life events with problem drinking in male current regular dri [file ADD-115-850-s001.docx]

| **Variables** | **Description** |
| --- | --- |
| **Drinking status** | |
| Abstainers^ | Past 12 months: Never drank alcohol.  In the past: Had not drunk ≥weekly. |
| Ex-regular drinkers* | Past 12 months: Never drank alcohol.  In the past: Had drunk ≥weekly. |
| Reduced-intake drinkers* | Past 12 months: Had drunk alcohol occasionally, at certain seasons, or monthly but less than weekly.  In the past: Had drunk ≥weekly. |
| Occasional drinkers | Past 12 months: Had drunk alcohol occasionally, at certain seasons, or monthly but less than weekly.  In the past: Had not drunk ≥weekly. |
| Current regular drinkers | Past 12 months: ≥Weekly (i.e., drank alcohol most weeks).  In the past: --N/A |
| **Drinking patterns** | |
| Drinking frequency | 1-2 days/week; 3-5 days/week; daily or almost every day. |
| Mean consumption^#^ | Weekly consumption in a typical drinking week (g/week): Calculated based on the beverage type, amount drunk (g of pure alcohol per typical drinking day) and frequency.  Consumption on special occasions (g/session): Calculated based on the beverage type and amount drunk (g of pure alcohol per session) on special occasions.  Consumption on the last drinking day (g/session): Calculated based on the beverage type and amount drunk (g of pure alcohol per session) on the last drinking day.  Alcohol content by volume (v/v) of each beverage type are assumed as the following: beer 4%, grape wine 12%, rice wine 15%, weak spirits 38% and strong spirits 53%. |
| Types of alcohol^#^ | Strong spirit (≥40% alcohol) only; weak spirit (<40% alcohol) only; wine (rice wine and grape wine) only; beer only; mixed (more than one type, available in questions related to special occasions and the last drinking day only). |
| Heavy episodic drinking (HED)^#^ | Men: consumption of >60 g of alcohol per session;  Women: consumption of >40 g of alcohol per session. |
| Problem drinking indicators | Reporting one or more of the following in the past month: (i) ever drinking in the morning; (ii) unable to work or do anything due to drinking; (iii) depressed, irritated or lost control due to drinking; (iv) couldn’t stop drinking; (v) had shakes when stopped drinking. |
| Flushing response | Experiencing hot flushes or dizziness soon after first mouthful or after drinking small amount of alcohol. |
| Drinking with/outside meals | Usually drink with meals; Usually drink between or after meals or no regular patterns. |
| **Problem drinking categories among current regular drinkers** | |
| Low-risk drinkers | Weekly alcohol intake <200g/week, no HED in a typical drinking week, no problem drinking indicator reported. |
| High-risk drinkers | Weekly alcohol intake ≥200g/week or HED in a typical drinking week; no problem drinking indicator reported. |
| Problem drinkers with 1 indicator | Reporting 1 problem drinking indicator. |
| Problem drinkers with 2+ indicators | Reporting 2 or more problem drinking indicators. |

**Table S1. Definitions of main alcohol drinking categories, pattern and problem drinking**

^Past-year abstainers.

*Ex-regular drinkers and reduced-intake drinkers were combined later in the present study.

^#^Data was available on a typical drinking day, on special occasions, and the last time the participants drank.

**Figure S1. Alcohol drinking status and problem drinking categorisation of study sample among CKB men**

Percentages represent proportion of the entire study sample of all men.

Abstainers were men who had not drunk alcohol in the past year and had not drunk ≥weekly previously; ex-regular drinkers were men who had drunk alcohol ≥weekly previously but had not drunk in the past year; reduced-intake drinkers were men who had drunk alcohol ≥weekly previously but had drunk less than weekly in the past year; occasional drinkers were men who reported drinking alcohol less than weekly in the past year and had not drunk alcohol ≥weekly previously. Low-risk drinkers were current regular drinkers (i.e., reported drinking ≥weekly in the past year) who drank <200g/week, with no HED in a typical drinking week or problem drinking indicator reported; high-risk drinkers were current regular drinkers who either drank at least 200g/week or engaged in HED in a typical drinking week, but with no problem drinking indicator reported; problem drinkers were current regular drinkers who reported at least one problem drinking indicator, and were further classified into “1 problem drinking indicator” and “2+ problem drinking indicators” according to the number of problem drinking indicators reported.

**Table S2. Details of main analytic models**

| **Exposure** | **Outcome** | **Analytic model and adjustment** | **Participants** |
| --- | --- | --- | --- |
| Experience of stressful life events  [reference group: no experience of the specified stressful life event] | Problem drinking, i.e., reporting at least 1 problem drinking indicator(s) | Logistic regression adjusted for age (in 5-year groups), study region (10 regions), highest education (4 groups: no formal education, primary school, middle or high school, technical school/college or above), household income (4 groups: <10,000, 10,000-19,999, 20,000-34,999, 35,000+ yuan/year), and smoking (4 groups: never, occasional, ex-regular, current regular smoker) | Current regular drinkers |
| Problem drinking categories  [reference group: low-risk drinkers] | Wellbeing-related measures:   - Poor self-reported heath - Life dissatisfaction - Sleep problems - Depression symptoms - Major depressive episode (MDE) - Anxiety symptom - General anxiety disorder (GAD) - Panic attacks - Phobia | Logistic regression adjusted for age (in 5-year groups), study region (10 regions), highest education (4 groups: no formal education, primary school, middle or high school, technical school/college or above), household income (4 groups: <10,000, 10,000-19,999, 20,000-34,999, 35,000+ yuan/year), smoking (4 groups: never, occasional, ex-regular, current regular smoker), marital status (4 groups: married, widowed, separated/divorced, never married), prior chronic diseases (yes vs.no), and body mass index (4 groups: <22, 22-24.9, 25-26.9, 27+ kg/m^2^) | Current regular drinkers |
| Problem drinking categories  [reference group: low-risk drinkers] | Mortality and hospitalised events:   - All-cause mortality - All hospitalisations - Fatal and non-fatal incidents due to external causes (ICD-10: V01-Y98) | Cox regression, stratified by age-at-risk (5-year intervals) and 10 regions, and adjusted for highest education (4 groups: no formal education, primary school, middle or high school, technical school/college or above), household income (4 groups: <10,000, 10,000-19,999, 20,000-34,999, 35,000+ yuan/year), smoking (4 groups: never, occasional, ex-regular, current regular smoker), fruit intake (daily vs. less than daily), physical activity (3 groups: <17.8, 17.8-28.7, 28.8+ metabolic equivalent of task hours [MET-h] per day)^a^, and body mass index (4 groups: <22, 22-24.9, 25-26.9, 27+ kg/m^2^) | Current regular drinkers without prior chronic diseases (coronary heart disease, stroke, transient ischemic attack, diabetes, cancer, cirrhosis/chronic hepatitis, tuberculosis, emphysema/bronchitis, peptic ulcer, gallstone/gallbladder disease, kidney disease and rheumatoid arthritis) |

^a^ Physical activity was estimated as the sum of metabolic equivalent of task hours per day based on questions on the usual type and duration of activities related to work, commuting, household chores, and leisure-time exercise in the past year. Questions on physical activity were adapted from validated questionnaires used in previous cohort studies including the European Prospective Investigation in Cancer and Nutrition^1^ and the Shanghai Women’s Health Study^2^, with some additional modifications after a CKB pilot study.

**Figure S2. Prevalence of problem drinking among male current regular drinkers, and of specific problem drinking indicators among problem drinkers, by age group**

Prevalences are adjusted for region.

**Figure S3. Prevalence of current regular drinking and problem drinking among all men**

Prevalences are adjusted for region in (a) and age in (b-c).

**Figure S4. Prevalence of problem drinking among male current regular drinkers, and of specific problem drinking indicators among problem drinkers, by ten study regions**

Prevalences are adjusted for age.

**Figure S5. Prevalence of problem drinking among male current regular drinkers, and of specific problem drinking indicators among problem drinkers, by rural and urban areas**

Prevalences are adjusted for age.

**Table S3. Drinking characteristics by problem drinking status in male current regular drinkers, by rural-urban regions**

|  |  |  |  | **Rural** | | | | | | | | | | |  | **Urban** | | | | | | | | | | | |
| --- | --- | --- | --- | --- | --- | --- | --- | --- | --- | --- | --- | --- | --- | --- | --- | --- | --- | --- | --- | --- | --- | --- | --- | --- | --- | --- | --- |
|  |  |  |  | **Low-risk drinkers** | |  | **High-risk drinkers** | |  | **Problem drinkers with 1 indicator** | |  | **Problem drinkers with 2+ indicators** | |  | | **Low-risk drinkers** | |  | **High-risk drinkers** | |  | **Problem drinkers with 1 indicator** | |  | **Problem drinkers with 2+ indicators** | |
| Number of men | . | . | . | 10023 |  | . | 13019 |  | . | 9239 |  | . | 2600 |  |  | | 15935 |  | . | 14235 |  | . | 4032 |  | . | 821 |  |
| **Drinking characteristics** |  |  |  |  |  |  |  |  |  |  |  |  |  |  |  | |  |  |  |  |  |  |  |  |  |  |  |
| Drank alcohol on the day of survey, % | . | . | . | 15.0 |  | . | 24.7 |  | . | 37.9 |  | . | 45.1 |  |  | | 6.5 |  | . | 11.6 |  | . | 19.3 |  | . | 25.7 |  |
| Daily drinking, % | . | . | . | 32.5 |  | . | 75.5 |  | . | 78.1 |  | . | 84.3 |  |  | | 39.3 |  | . | 76.1 |  | . | 75.8 |  | . | 83.9 |  |
| Age started regular drinking, mean(SD) | . | . | . | 30.7 | (12.4) | . | 27.0 | (10.1) | . | 26.4 | (10.5) | . | 25.2 | (9.4) |  | | 31.7 | (12.0) | . | 27.4 | (8.9) | . | 28.9 | (10.8) | . | 27.1 | (9.0) |
| Drinking with meals, % | . | . | . | 81.0 |  | . | 85.6 |  | . | 81.3 |  | . | 80.7 |  |  | | 89.9 |  | . | 88.4 |  | . | 88.7 |  | . | 87.3 |  |
| Flushing response^*^, % | . | . | . | 26.6 |  | . | 17.2 |  | . | 16.2 |  | . | 15.7 |  |  | | 21.0 |  | . | 12.1 |  | . | 13.1 |  | . | 12.9 |  |
| **Typical drinking week** |  |  |  |  |  |  |  |  |  |  |  |  |  |  |  | |  |  |  |  |  |  |  |  |  |  |  |
| Mean weekly consumption, g/week(SD) | . | . | . | 99.1 | (56.6) | . | 402.1 | (218.5) | . | 426.8 | (288.8) | . | 558.7 | (348.6) |  | | 98.7 | (56.4) | . | 367.2 | (198.6) | . | 303.0 | (227.4) | . | 394.9 | (298.8) |
| Heavy episodic drinking (HED), % | . | . | . | 0.0 |  | . | 66.9 |  | . | 57.7 |  | . | 72.6 |  |  | | 0.0 |  | . | 56.1 |  | . | 36.1 |  | . | 51.1 |  |
| Heavy drinking (200+g/week), % | . | . | . | 0.0 |  | . | 88.2 |  | . | 74.9 |  | . | 86.4 |  |  | | 0.0 |  | . | 89.2 |  | . | 63.1 |  | . | 75.7 |  |
| HED and heavy drinking, % | . | . | . | 0.0 |  | . | 55.2 |  | . | 54.3 |  | . | 69.4 |  |  | | 0.0 |  | . | 45.3 |  | . | 33.8 |  | . | 49.6 |  |
| Beverage types consumed, % |  |  |  |  |  |  |  |  |  |  |  |  |  |  |  | |  |  |  |  |  |  |  |  |  |  |  |
| Strong spirit (>=40% alcohol) only | . | . | . | 45.2 |  | . | 70.6 |  | . | 64.8 |  | . | 68.6 |  |  | | 17.3 |  | . | 44.6 |  | . | 42.9 |  | . | 52.1 |  |
| Weak spirit (<40% alcohol) only | . | . | . | 28.4 |  | . | 23.6 |  | . | 25.8 |  | . | 23.9 |  |  | | 19.2 |  | . | 22.3 |  | . | 15.6 |  | . | 14.1 |  |
| Beer only | . | . | . | 15.4 |  | . | 2.3 |  | . | 3.4 |  | . | 2.3 |  |  | | 39.4 |  | . | 21.3 |  | . | 25.2 |  | . | 22.7 |  |
| Rice wine or grape wine only | . | . | . | 11.0 |  | . | 3.5 |  | . | 6.0 |  | . | 5.2 |  |  | | 24.0 |  | . | 11.8 |  | . | 16.2 |  | . | 11.1 |  |
| **Special occasion** |  |  |  |  |  |  |  |  |  |  |  |  |  |  |  | |  |  |  |  |  |  |  |  |  |  |  |
| Mean consumption per session, g/session(SD) | . | . | . | 108.4 | (90.4) | . | 169.0 | (113.4) | . | 166.0 | (113.2) | . | 202.9 | (136.3) |  | | 107.2 | (83.5) | . | 173.1 | (108.7) | . | 155.7 | (109.0) | . | 190.9 | (127.8) |
| HED, % | . | . | . | 67.9 |  | . | 93.6 |  | . | 90.6 |  | . | 94.5 |  |  | | 70.2 |  | . | 93.8 |  | . | 85.4 |  | . | 92.2 |  |
| **Last time drinking** |  |  |  |  |  |  |  |  |  |  |  |  |  |  |  | |  |  |  |  |  |  |  |  |  |  |  |
| Mean consumption per day, g/day(SD) | . | . | . | 30.6 | (22.1) | . | 55.6 | (35.6) | . | 54.5 | (37.7) | . | 62.2 | (44.3) |  | | 34.6 | (29.1) | . | 66.4 | (46.1) | . | 58.2 | (45.1) | . | 72.3 | (55.9) |
| HED, % | . | . | . | 8.5 |  | . | 36.6 |  | . | 34.3 |  | . | 41.3 |  |  | | 14.5 |  | . | 45.9 |  | . | 40.8 |  | . | 51.7 |  |

SD, standard deviation; HED, heavy episodic drinking.

Prevalences and means are adjusted for age group.

Associations between problem drinking categories and drinking characteristic variables evaluated with a chi-square test for association: p<0.0001 across all variables

Low-risk drinkers were current regular drinkers who drank <200g/week, with no HED in a typical drinking week or problem drinking indicator reported; high-risk drinkers were current regular drinkers who either drank at least 200g/week or engaged in HED in a typical drinking week, but with no problem drinking indicator reported; problem drinkers were current regular drinkers who reported at least one problem drinking indicator, and were further classified into “1 problem drinking indicator” and “2+ problem drinking indicators” according to the number of problem drinking indicators reported.

^*^Experiencing hot flushes or dizziness soon after first mouthful or after drinking small amount of alcohol.

**Table S4. Drinking characteristics by specific problem drinking indicators in male current regular drinkers**

|  | | | | **Unable to work** | |  | **Negative emotions** | |  | **Unable to stop** | |  | **Had shakes** | |  | **Morning drinking** | |
| --- | --- | --- | --- | --- | --- | --- | --- | --- | --- | --- | --- | --- | --- | --- | --- | --- | --- |
| Number of men | . | . | . | 2428 |  | . | 1102 |  | . | 7796 |  | . | 281 |  | . | 9171 |  |
| **Drinking characteristics** | | | | | | | | | | | | | | | | | |
| Drank alcohol on the day of survey, % | . | . | . | 22.5 |  | . | 28.0 |  | . | 26.3 |  | . | 32.7 |  | . | 41.2 |  |
| Daily drinking, % | . | . | . | 66.1 |  | . | 71.9 |  | . | 86.6 |  | . | 78.4 |  | . | 81.4 |  |
| Age started regular drinking, mean (SD) | . | . | . | 27.2 | (9.4) | . | 26.6 | (9.0) | . | 26.7 | (9.5) | . | 27.3 | (10.4) | . | 27.3 | (11.2) |
| Drinking with meals, % | . | . | . | 85.4 |  | . | 85.1 |  | . | 85.9 |  | . | 82.2 |  | . | 80.1 |  |
| Flushing response*, % | . | . | . | 16.4 |  | . | 17.9 |  | . | 14.9 |  | . | 14.7 |  | . | 14.4 |  |
| Reporting 2+ problem drinking indicators, % |  |  |  | 47.1 |  |  | 73.2 |  |  | 36.8 |  |  | 81.9 |  |  | 26.8 |  |
| **Typical drinking week** | | | | | | | | | | | | | | | | | |
| Mean weekly consumption, g/week (SD) | . | . | . | 381.6 | (304.7) | . | 437.7 | (345.8) | . | 428.2 | (285.1) | . | 499.8 | (411.8) | . | 440.9 | (334.7) |
| Heavy episodic drinking (HED), % | . | . | . | 52.4 |  | . | 56.1 |  | . | 53.7 |  | . | 54.8 |  | . | 55.4 |  |
| Heavy drinking (200+g/week), % | . | . | . | 65.2 |  | . | 72.2 |  | . | 79.1 |  | . | 70.9 |  | . | 74.8 |  |
| HED and heavy drinking, % | . | . | . | 46.5 |  | . | 52.5 |  | . | 52.5 |  | . | 52.7 |  | . | 53.3 |  |
| Beverage types consumed, % | | | | | | | | | | | | | | | | | |
| Strong spirits (≥40% alcohol) only | . | . | . | 49.3 |  | . | 50.6 |  | . | 56.3 |  | . | 53.9 |  | . | 55.6 |  |
| Weak spirits (<40% alcohol) only | . | . | . | 23.0 |  | . | 23.8 |  | . | 23.1 |  | . | 20.1 |  | . | 24.6 |  |
| Beer only | . | . | . | 15.3 |  | . | 15.4 |  | . | 11.0 |  | . | 13.0 |  | . | 8.9 |  |
| Rice wine or grape wine only | . | . | . | 12.4 |  | . | 10.1 |  | . | 9.6 |  | . | 13.0 |  | . | 11.0 |  |
| **Special occasion** | | | | | | | | | | | | | | | | | |
| Mean consumption per session, g/session (SD) | . | . | . | 200.8 | (141.5) | . | 212.1 | (146.9) | . | 180.4 | (117.6) | . | 198.9 | (135.7) | . | 171.9 | (111.7) |
| HED, % | . | . | . | 91.6 |  | . | 92.2 |  | . | 93.0 |  | . | 88.1 |  | . | 88.8 |  |
| **Last time drinking** | | | | | | | | | | | | | | | | | |
| Mean consumption per session, g/session (SD) | . | . | . | 70.1 | (54.0) | . | 72.9 | (56.6) | . | 59.4 | (39.1) | . | 69.1 | (53.5) | . | 58.0 | (40.5) |
| HED, % | . | . | . | 45.0 |  | . | 47.0 |  | . | 41.3 |  | . | 47.9 |  | . | 41.4 |  |

SD, standard deviation; HED, heavy episodic drinking.

Prevalences and means are adjusted for age group and region.

The problem drinking indicators are not mutually exclusive.

*Experiencing hot flushes or dizziness soon after first mouthful or after drinking small amount of alcohol.

**Table S5. Cross-sectional associations of stressful life events with problem drinking in male current regular drinkers, by rural and urban regions**

|  | |  | | **Rural** | | | | | | | | | | | | |  | **Urban** | | | | | | | | | | | | |
| --- | --- | --- | --- | --- | --- | --- | --- | --- | --- | --- | --- | --- | --- | --- | --- | --- | --- | --- | --- | --- | --- | --- | --- | --- | --- | --- | --- | --- | --- | --- |
|  | |  | | **Total current regular drinkers**  **N** | |  | | | | **Problem drinkers^a^**  **N (%)** |  | | **Adjusted OR (95% CI) for problem drinking** | | ***p-value*** | |  | **Total current regular drinker**  **N** | |  | | | | **Problem drinkers^a^**  **N (%)** |  | | **Adjusted OR (95% CI) for problem drinking** | | ***p-value*** | |
| **Family-related events** | | | | | | | | | | | | | | | | | | | | | | | | | | | | | | |
| Divorce or separation | | | | | | | | | | | | | | | | | | | | | | | | | | | | | | |
| No | | . | | 34818 | | . | | 11820 (33.9%) | | | . | | 1.00 | |  | | | 34883 | | . | | 4819 (13.8%) | | | . | | 1.00 | |  | |
| Yes | | . | | 63 | | . | | 19 (30.2%) | | | . | | 0.85 (0.49-1.46) | | *0.5544* | | | 140 | | . | | 34 (24.3%) | | | . | | 1.65 (1.10-2.48) | | *0.0163* | |
| Family conflict | | | | | | | | | | | | | | | | | | | | | | | | | | | | | | |
| No | | . | | 34628 | | . | | 11739 (33.9%) | | | . | | 1.00 | |  | | | 34789 | | . | | 4801 (13.8%) | | | . | | 1.00 | |  | |
| Yes | | . | | 253 | | . | | 100 (39.5%) | | | . | | 1.32 (1.02-1.71) | | *0.0320* | | | 234 | | . | | 52 (22.2%) | | | . | | 1.86 (1.34-2.59) | | *0.0002* | |
| Death of spouse | | | | | | | | | | | | | | | | | | | | | | | | | | | | | | |
| No | | . | | 34690 | | . | | 11768 (33.9%) | | | . | | 1.00 | |  | | | 34860 | | . | | 4828 (13.8%) | | | . | | 1.00 | |  | |
| Yes | | . | | 191 | | . | | 71 (37.2%) | | | . | | 1.10 (0.82-1.49) | | *0.5143* | | | 163 | | . | | 25 (15.3%) | | | . | | 0.94 (0.60-1.46) | | *0.7746* | |
| Death or major illness of other family member | | | | | | | | | | | | | | | | | | | | | | | | | | | | | | |
| No | | . | | 33447 | | . | | 11276 (33.7%) | | | . | | 1.00 | |  | | | 33233 | | . | | 4595 (13.8%) | | | . | | 1.00 | |  | |
| Yes | | . | | 1434 | | . | | 563 (39.3%) | | | . | | 1.23 (1.10-1.37) | | *0.0002* | | | 1790 | | . | | 258 (14.4%) | | | . | | 1.15 (1.00-1.33) | | *0.0511* | |
| Any family-related events | | | | | | | | | | | | | | | | | | | | | | | | | | | | | | |
| No | | . | | 32992 | | . | | 11110 (33.7%) | | | . | | 1.00 | |  | | | 32756 | | . | | 4496 (13.7%) | | | . | | 1.00 | |  | |
| Yes | | . | | 1889 | | . | | 729 (38.6%) | | | . | | 1.21 (1.09-1.33) | | *0.0002* | | | 2267 | | . | | 357 (15.7%) | | | . | | 1.23 (1.09-1.39) | | *0.0010* | |
| **Finance-related events** | | | | | | | | | | | | | | | | | | | | | | | | | | | | | | |
| Job loss or retirement | | | | | |  | |  | | |  | |  | |  | | |  | |  | |  | | |  | |  | |  | |
| No | | . | | 34847 | | . | | 11825 (33.9%) | | | . | | 1.00 | |  | | | 34740 | | . | | 4797 (13.8%) | | | . | | 1.00 | |  | |
| Yes | | . | | 34 | | . | | 14 (41.2%) | | | . | | 1.63 (0.81-3.26) | | *0.1684* | | | 283 | | . | | 56 (19.8%) | | | . | | 1.30 (0.95-1.78) | | *0.0968* | |
| Bankruptcy | | | | | | | | | | | | | | | | | | | | | | | | | | | | | | |
| No | | . | | 34697 | | . | | 11760 (33.9%) | | | . | | 1.00 | |  | | | 34986 | | . | | 4848 (13.9%) | | | . | | 1.00 | |  | |
| Yes | | . | | 184 | | . | | 79 (42.9%) | | | . | | 1.53 (1.14-2.07) | | *0.0050* | | | 37 | | . | | 5 (13.5%) | | | . | | 1.05 (0.39-2.77) | | *0.9287* | |
| Loss of income or debt | | | | | | | | | | | | | | | | | | | | | | | | | | | | | | |
| No | | . | | 34724 | | . | | 11756 (33.9%) | | | . | | 1.00 | |  | | | 34868 | | . | | 4821 (13.8%) | | | . | | 1.00 | |  | |
| Yes | | . | | 157 | | . | | 83 (52.9%) | | | . | | 2.13 (1.55-2.93) | | *<.0001* | | | 155 | | . | | 32 (20.6%) | | | . | | 1.37 (0.90-2.09) | | *0.1383* | |
| Any finance-related events | | | | | | | | | | | | | | | | | | | | | | | | | | | | | | |
| No | | . | | 34521 | | . | | 11671 (33.8%) | | | . | | 1.00 | |  | | | 34578 | | . | | 4770 (13.8%) | | | . | | 1.00 | |  | |
| Yes | | . | | 360 | | . | | 168 (46.7%) | | | . | | 1.76 (1.42-2.18) | | *<.0001* | | | 445 | | . | | 83 (18.7%) | | | . | | 1.24 (0.96-1.61) | | *0.0952* | |
| **Injury-related events** | | | | | | | | | | | | | | | | | | | | | | | | | | | | | | |
| Violence | | | | | | | | | | | | | | | | | | | | | | | | | | | | | | |
| No | | . | | 34807 | | . | | 11803 (33.9%) | | | . | | 1.00 | |  | | | 34989 | | . | | 4850 (13.9%) | | | . | | 1.00 | |  | |
| Yes | | . | | 74 | | . | | 36 (48.6%) | | | . | | 1.93 (1.22-3.07) | | *0.0053* | | | 34 | | . | | 3 (8.8%) | | | . | | 0.78 (0.23-2.68) | | *0.6948* | |
| Major injury or traffic accident | | | | | | | | | | | | | | | | | | | | | | | | | | | | | | |
| No | | . | | 34595 | | . | | 11732 (33.9%) | | | . | | 1.00 | |  | | | 34808 | | . | | 4834 (13.9%) | | | . | | 1.00 | |  | |
| Yes | | . | | 286 | | . | | 107 (37.4%) | | | . | | 1.23 (0.96-1.56) | | *0.1023* | | | 215 | | . | | 19 (8.8%) | | | . | | 0.92 (0.56-1.49) | | *0.7286* | |
| Natural disaster | | | | | | | | | | | | | | | | | | | | | | | | | | | | | | |
| No | | . | | 34848 | | . | | 11826 (33.9%) | | | . | | 1.00 | |  | | | 35000 | | . | | 4853 (13.9%) | | | . | | 1.00 | |  | |
| Yes | | . | | 33 | | . | | 13 (39.4%) | | | . | | 1.48 (0.73-3.01) | | *0.2786* | | | 23 | | . | | 0 (0.0%) | | | . | | -- | | *--* | |
| Any injury or disaster events | | | | | | | | | | | | | | | | | | | | | | | | | | | | | | |
| No | | . | | 34490 | | . | | 11685 (33.9%) | | | . | | 1.00 | |  | | | 34754 | | . | | 4831 (13.9%) | | | . | | 1.00 | |  | |
| Yes | | . | | 391 | | . | | 154 (39.4%) | | | . | | 1.35 (1.10-1.66) | | *0.0047* | | | 269 | | . | | 22 (8.2%) | | | . | | 0.80 (0.51-1.26) | | *0.3383* | |
| **Any major stressful life event** | | | | | | | | | | | | | | | | | | | | | | | | | | | | | | |
| No | | . | | 32363 | | . | | 10855 (33.5%) | | | . | | 1.00 | |  | | | 32146 | | . | | 4411 (13.7%) | | | . | | 1.00 | |  | |
| Yes | | . | | 2518 | | . | | 984 (39.1%) | | | . | | 1.26 (1.16-1.38) | | *<.0001* | | | 2877 | | . | | 442 (15.4%) | | | . | | 1.19 (1.07-1.33) | | *0.0019* | |

OR, Odds ratio; CI, Confidence interval.

All analyses were adjusted for age, study sites, education, income and smoking status. All p-values are for Wald chi-square test.

^a^ Reporting one or more in the past month of: drinking in the morning, unable to work or do anything due to drinking; depressed irritated or lost control due to drinking; couldn't stop drinking; had shakes when stopped drinking.

**Figure S6. Cross-sectional associations of problem drinking with wellbeing in male current regular drinkers, by rural and urban regions**

ORs were adjusted for age group, region, education, income, marital status, prior chronic diseases, smoking and BMI. Each solid square represents an OR. 95% CIs are plotted using floating standard errors to allow for comparison between any two categories. The size of each box is inversely proportional to the ‘floated’ variance of the log OR in each group and the error bars indicate the group-specific 95% CI. OR, odds ratio; CI, confidence interval; BMI, body mass index; HED, heavy episodic drinking. Low-risk drinkers were current regular drinkers who drank <200g/week, with no HED in a typical drinking week or problem drinking indicator reported; high-risk drinkers were current regular drinkers who either drank at least 200g/week or engaged in HED in a typical drinking week, but with no problem drinking indicator reported; problem drinkers were current regular drinkers who reported at least one problem drinking indicator, and were further classified into “1 problem drinking indicator” and “2+ problem drinking indicators” according to the number of problem drinking indicators reported. General anxiety disorder was not shown in this figure due to the limited number of events.

**Table S6. Adjusted odds ratios (ORs) associated with an increase in the number of problem drinking indicators in men with at least one problem drinking indicator**

|  | | **Cases N** |  | **Non-cases N** |  | **Adjusted OR (95% CI)** |  | ***p-value*** |
| --- | --- | --- | --- | --- | --- | --- | --- | --- |
| **Poor self-reported health** | . | 1313 | . | 15379 | . | 1.28 (1.17-1.40) | . | *<.0001* |
| **Life dissatisfaction** | . | 748 | . | 15944 | . | 1.22 (1.08-1.38) | . | *0.0011* |
| **Sleep problems^a^** | . | 2965 | . | 13727 | . | 1.08 (1.01-1.16) | . | *0.0333* |
| **Depression symptoms^b^** | . | 571 | . | 16121 | . | 1.40 (1.24-1.58) | . | *<.0001* |
| **Major depressive episode^c^** | . | 91 | . | 16601 | . | 1.45 (1.11-1.91) | . | *0.0073* |
| **Anxiety symptom** | . | 111 | . | 16581 | . | 1.39 (1.07-1.80) | . | *0.0126* |
| **General anxiety disorder^d^** | . | 49 | . | 16643 | . | 1.24 (0.82-1.89) | . | *0.3077* |
| **Panic** | . | 166 | . | 16526 | . | 1.53 (1.25-1.88) | . | *<.0001* |
| **Phobic** | . | 86 | . | 16606 | . | 0.99 (0.67-1.45) | . | *0.9434* |

OR, odds ratio; CI, confidence interval; BMI, body mass index; CIDI-SF, composite international diagnostic interview short-form.

ORs were adjusted for age group, region, education, income, marital status, prior chronic diseases, smoking and BMI.

^a^ Reporting one or more of the following for ≥ 3 days each week in the past month: delayed or fitful sleep; waking up too early; needing medicine to help sleep; having difficulty staying alert during daytime.

^b^ Reporting one or more of the following for ≥ 2 weeks in the past 12 months: feeling sad or depressed; loss of interest; loss of appetite; feeling worthless.

^c^ Assessed by the CIDI-SF (A).

^d^ Assessed by the CIDI-SF (B).

**Table S7. Cross-sectional associations of problem drinking with sleep problems in male current regular drinkers**

|  | | **Cases N** |  | **Non-cases N** |  | **Adjusted OR (95% CI)** |  | ***p-heterogeneity*** |
| --- | --- | --- | --- | --- | --- | --- | --- | --- |
| **Delayed/fitful sleep** | | | | | | | | |
| Low-risk drinkers | . | 2082 | . | 23876 | . | 1.00 (0.95-1.05) | . | *.* |
| High-risk drinkers | . | 2221 | . | 25033 | . | 1.04 (1.00-1.09) | . | *.* |
| 1 problem drinking indicator | . | 1469 | . | 11802 | . | 1.28 (1.21-1.35) | . | *.* |
| 2+ problem drinking indicators | . | 426 | . | 2995 | . | 1.39 (1.25-1.54) | . | *<.0001* |
| **Early-morning awakening** | | | | | | | | |
| Low-risk drinkers | . | 1925 | . | 24033 | . | 1.00 (0.95-1.05) | . | *.* |
| High-risk drinkers | . | 2221 | . | 25033 | . | 1.14 (1.09-1.20) | . | *.* |
| 1 problem drinking indicator | . | 1517 | . | 11754 | . | 1.41 (1.33-1.49) | . | *.* |
| 2+ problem drinking indicators | . | 439 | . | 2982 | . | 1.60 (1.45-1.78) | . | *<.0001* |
| **Sleep medication** | | | | | | | | |
| Low-risk drinkers | . | 161 | . | 25797 | . | 1.00 (0.85-1.18) | . | *.* |
| High-risk drinkers | . | 124 | . | 27130 | . | 0.89 (0.75-1.07) | . | *.* |
| 1 problem drinking indicator | . | 92 | . | 13179 | . | 1.38 (1.12-1.71) | . | *.* |
| 2+ problem drinking indicators | . | 33 | . | 3388 | . | 1.87 (1.32-2.66) | . | *0.0002* |
| **Difficulty in staying alert during daytime** | | | | | | | | |
| Low-risk drinkers | . | 303 | . | 25655 | . | 1.00 (0.89-1.13) | . | *.* |
| High-risk drinkers | . | 357 | . | 26897 | . | 1.07 (0.96-1.19) | . | *.* |
| 1 problem drinking indicator | . | 246 | . | 13025 | . | 1.56 (1.37-1.78) | . | *.* |
| 2+ problem drinking indicators | . | 95 | . | 3326 | . | 2.02 (1.64-2.49) | . | *<.0001* |

OR, odds ratio; CI, confidence interval; BMI, body mass index; HED, heavy episodic drinking.

Models were adjusted for age group, region, education, income, marital status, prior chronic diseases, smoking and BMI.

Low-risk drinkers were current regular drinkers who drank <200g/week, with no HED in a typical drinking week or problem drinking indicator reported; high-risk drinkers were current regular drinkers who either drank at least 200g/week or engaged in HED in a typical drinking week, but with no problem drinking indicator reported; problem drinkers were current regular drinkers who reported at least one problem drinking indicator, and were further classified into “1 problem drinking indicator” and “2+ problem drinking indicators” according to the number of problem drinking indicators reported.

**Table S8. Cross-sectional associations of problem drinking indicators with wellbeing-related measures in male current regular drinkers**

|  | | | **Morning drinking** | | | | |  | | **Unable to stop** | | | | |  | | **Unable to work** | | | | |  | | **Negative emotions** | | | | |  | | **Having shakes** | | | | |
| --- | --- | --- | --- | --- | --- | --- | --- | --- | --- | --- | --- | --- | --- | --- | --- | --- | --- | --- | --- | --- | --- | --- | --- | --- | --- | --- | --- | --- | --- | --- | --- | --- | --- | --- | --- |
|  | | | **Non- cases N** |  | **Cases N** |  | **OR (95% CI)** |  | | **Non- cases N** |  | **Cases N** |  | **OR (95% CI)** |  | | **Non- cases N** |  | **Cases N** |  | **OR (95% CI)** |  | | **Non- cases N** |  | **Cases N** |  | **OR (95% CI)** |  | | **Non- cases N** |  | **Cases N** |  | **OR (95% CI)** |
| **Poor self-reported health** | | | | | | | | | | | | | | | | | | | | | | | | | | | | | | | | | | | |
| Low-risk drinkers | . | . | 24478 | . | 1480 | . | 1.00 (0.94-1.06) | . | . | 24478 | . | 1480 | . | 1.00 (0.94-1.06) | . | . | 24478 | . | 1480 | . | 1.00 (0.94-1.06) | . | . | 24478 | . | 1480 | . | 1.00 (0.94-1.06) | . | . | 24478 | . | 1480 | . | 1.00 (0.94-1.06) |
| High-risk drinkers | . | . | 25651 | . | 1603 | . | 0.97 (0.92-1.02) | . | . | 25651 | . | 1603 | . | 0.97 (0.92-1.02) | . | . | 25651 | . | 1603 | . | 0.97 (0.92-1.02) | . | . | 25651 | . | 1603 | . | 0.97 (0.92-1.02) | . | . | 25651 | . | 1603 | . | 0.97 (0.92-1.02) |
| Specified indicator* | . | . | 8540 | . | 631 | . | 1.14 (1.05-1.25) | . | . | 7085 | . | 711 | . | 1.21 (1.11-1.31) | . | . | 2173 | . | 255 | . | 1.63 (1.42-1.86) | . | . | 965 | . | 137 | . | 1.78 (1.47-2.14) | . | . | 247 | . | 34 | . | 1.87 (1.29-2.71) |
| Other indicator(s)^ | . | . | 6839 | . | 682 | . | 1.22 (1.12-1.33) | . | . | 8294 | . | 602 | . | 1.16 (1.06-1.27) | . | . | 13206 | . | 1058 | . | 1.10 (1.03-1.18) | . | . | 14414 | . | 1176 | . | 1.14 (1.06-1.21) | . | . | 15132 | . | 1279 | . | 1.17 (1.10-1.25) |
|  | . | . |  | . |  | . | *p-het = <.0001* | . | . |  | . |  | . | *p-het = <.0001* | . | . |  | . |  | . | *p-het = <.0001* | . | . |  | . |  | . | *p-het = <.0001* | . | . |  | . |  | . | *p-het = <.0001* |
| **Life dissatisfaction** | | | | | | | | | | | | | | | | | | | | | | | | | | | | | | | | | | | |
| Low-risk drinkers | . | . | 24821 | . | 1137 | . | 1.00 (0.94-1.07) | . | . | 24821 | . | 1137 | . | 1.00 (0.94-1.07) | . | . | 24821 | . | 1137 | . | 1.00 (0.94-1.06) | . | . | 24821 | . | 1137 | . | 1.00 (0.94-1.07) | . | . | 24821 | . | 1137 | . | 1.00 (0.94-1.06) |
| High-risk drinkers | . | . | 26118 | . | 1136 | . | 1.00 (0.94-1.07) | . | . | 26118 | . | 1136 | . | 1.00 (0.94-1.07) | . | . | 26118 | . | 1136 | . | 1.00 (0.94-1.06) | . | . | 26118 | . | 1136 | . | 1.00 (0.94-1.07) | . | . | 26118 | . | 1136 | . | 1.00 (0.94-1.07) |
| Specified indicator* | . | . | 8827 | . | 344 | . | 1.24 (1.10-1.40) | . | . | 7446 | . | 350 | . | 1.30 (1.15-1.46) | . | . | 2248 | . | 180 | . | 1.54 (1.31-1.82) | . | . | 1002 | . | 100 | . | 1.85 (1.49-2.30) | . | . | 256 | . | 25 | . | 2.20 (1.42-3.42) |
| Other indicator(s)^ | . | . | 7117 | . | 404 | . | 1.34 (1.20-1.49) | . | . | 8498 | . | 398 | . | 1.29 (1.16-1.44) | . | . | 13696 | . | 568 | . | 1.23 (1.12-1.35) | . | . | 14942 | . | 648 | . | 1.23 (1.13-1.35) | . | . | 15688 | . | 723 | . | 1.28 (1.17-1.38) |
|  | . | . |  | . |  | . | *p-het = <.0001* | . | . |  | . |  | . | *p-het = <.0001* | . | . |  | . |  | . | *p-het = <.0001* | . | . |  | . |  | . | *p-het = <.0001* | . | . |  | . |  | . | *p-het = <.0001* |
| **Sleep problems^a^** | | | | | | | | | | | | | | | | | | | | | | | | | | | | | | | | | | | |
| Low-risk drinkers | . | . | 22803 | . | 3155 | . | 1.00 (0.96-1.04) | . | . | 22803 | . | 3155 | . | 1.00 (0.96-1.04) | . | . | 22803 | . | 3155 | . | 1.00 (0.96-1.04) | . | . | 22803 | . | 3155 | . | 1.00 (0.96-1.04) | . | . | 22803 | . | 3155 | . | 1.00 (0.96-1.04) |
| High-risk drinkers | . | . | 23751 | . | 3503 | . | 1.08 (1.04-1.12) | . | . | 23751 | . | 3503 | . | 1.08 (1.04-1.12) | . | . | 23751 | . | 3503 | . | 1.08 (1.04-1.12) | . | . | 23751 | . | 3503 | . | 1.08 (1.04-1.12) | . | . | 23751 | . | 3503 | . | 1.08 (1.04-1.12) |
| Specified indicator* | . | . | 7594 | . | 1577 | . | 1.24 (1.17-1.32) | . | . | 6409 | . | 1387 | . | 1.31 (1.23-1.39) | . | . | 1942 | . | 486 | . | 1.67 (1.51-1.85) | . | . | 849 | . | 253 | . | 1.98 (1.72-2.29) | . | . | 215 | . | 66 | . | 1.96 (1.48-2.60) |
| Other indicator(s)^ | . | . | 6133 | . | 1388 | . | 1.44 (1.36-1.53) | . | . | 7318 | . | 1578 | . | 1.36 (1.28-1.44) | . | . | 11785 | . | 2479 | . | 1.27 (1.22-1.34) | . | . | 12878 | . | 2712 | . | 1.29 (1.23-1.35) | . | . | 13512 | . | 2899 | . | 1.32 (1.27-1.38) |
|  | . | . |  | . |  | . | *p-het = <.0001* | . | . |  | . |  | . | *p-het = <.0001* | . | . |  | . |  | . | *p-het = <.0001* | . | . |  | . |  | . | *p-het = <.0001* | . | . |  | . |  | . | *p-het = <.0001* |
| **Depression symptoms^b^** | | | | | | | | | | | | | | | | | | | | | | | | | | | | | | | | | | | |
| Low-risk drinkers | . | . | 25496 | . | 462 | . | 1.00 (0.91-1.10) | . | . | 25496 | . | 462 | . | 1.00 (0.91-1.10) | . | . | 25496 | . | 462 | . | 1.00 (0.91-1.10) | . | . | 25496 | . | 462 | . | 1.00 (0.91-1.10) | . | . | 25496 | . | 462 | . | 1.00 (0.91-1.10) |
| High-risk drinkers | . | . | 26774 | . | 480 | . | 0.95 (0.87-1.04) | . | . | 26774 | . | 480 | . | 0.95 (0.87-1.04) | . | . | 26774 | . | 480 | . | 0.95 (0.87-1.04) | . | . | 26774 | . | 480 | . | 0.95 (0.87-1.04) | . | . | 26774 | . | 480 | . | 0.95 (0.87-1.04) |
| Specified indicator* | . | . | 8899 | . | 272 | . | 1.43 (1.25-1.63) | . | . | 7527 | . | 269 | . | 1.73 (1.52-1.97) | . | . | 2289 | . | 139 | . | 2.38 (1.99-2.84) | . | . | 1003 | . | 99 | . | 3.74 (3.01-4.63) | . | . | 261 | . | 20 | . | 3.24 (2.04-5.16) |
| Other indicator(s)^ | . | . | 7222 | . | 299 | . | 1.91 (1.69-2.15) | . | . | 8594 | . | 302 | . | 1.60 (1.42-1.81) | . | . | 13832 | . | 432 | . | 1.49 (1.35-1.66) | . | . | 15118 | . | 472 | . | 1.47 (1.33-1.62) | . | . | 15860 | . | 551 | . | 1.63 (1.49-1.79) |
|  | . | . |  | . |  | . | *p-het = <.0001* | . | . |  | . |  | . | *p-het = <.0001* | . | . |  | . |  | . | *p-het = <.0001* | . | . |  | . |  | . | *p-het = <.0001* | . | . |  | . |  | . | *p-het = <.0001* |
| **Major depressive episode^c^** | | | | | | | | | | | | | | | | | | | | | | | | | | | | | | | | | | | |
| Low-risk drinkers | . | . | 25865 | . | 93 | . | 1.00 (0.81-1.24) | . | . | 25865 | . | 93 | . | 1.00 (0.81-1.24) | . | . | 25865 | . | 93 | . | 1.00 (0.81-1.24) | . | . | 25865 | . | 93 | . | 1.00 (0.81-1.24) | . | . | 25865 | . | 93 | . | 1.00 (0.81-1.24) |
| High-risk drinkers | . | . | 27165 | . | 89 | . | 0.90 (0.73-1.10) | . | . | 27165 | . | 89 | . | 0.90 (0.73-1.11) | . | . | 27165 | . | 89 | . | 0.90 (0.73-1.10) | . | . | 27165 | . | 89 | . | 0.89 (0.73-1.10) | . | . | 27165 | . | 89 | . | 0.90 (0.73-1.11) |
| Specified indicator* | . | . | 9123 | . | 48 | . | 1.18 (0.86-1.61) | . | . | 7754 | . | 42 | . | 1.31 (0.95-1.81) | . | . | 2409 | . | 19 | . | 1.52 (0.95-2.41) | . | . | 1084 | . | 18 | . | 3.05 (1.88-4.93) | . | . | 279 | . | 2 | . | 1.41 (0.35-5.71) |
| Other indicator(s)^ | . | . | 7478 | . | 43 | . | 1.32 (0.97-1.80) | . | . | 8847 | . | 49 | . | 1.19 (0.89-1.60) | . | . | 14192 | . | 72 | . | 1.18 (0.92-1.52) | . | . | 15517 | . | 73 | . | 1.07 (0.84-1.37) | . | . | 16322 | . | 89 | . | 1.24 (1.00-1.55) |
|  | . | . |  | . |  | . | *p-het = 0.1778* | . | . |  | . |  | . | *p-het = 0.1797* | . | . |  | . |  | . | *p-het = 0.1302* | . | . |  | . |  | . | *p-het = <.0001* | . | . |  | . |  | . | *p-het = 0.1943* |
| **Anxiety symptom** | | | | | | | | | | | | | | | | | | | | | | | | | | | | | | | | | | | |
| Low-risk drinkers | . | . | 25883 | . | 75 | . | 1.00 (0.79-1.27) | . | . | 25883 | . | 75 | . | 1.00 (0.79-1.27) | . | . | 25883 | . | 75 | . | 1.00 (0.79-1.27) | . | . | 25883 | . | 75 | . | 1.00 (0.79-1.27) | . | . | 25883 | . | 75 | . | 1.00 (0.79-1.27) |
| High-risk drinkers | . | . | 27156 | . | 98 | . | 0.99 (0.81-1.21) | . | . | 27156 | . | 98 | . | 0.99 (0.81-1.21) | . | . | 27156 | . | 98 | . | 0.99 (0.81-1.20) | . | . | 27156 | . | 98 | . | 0.98 (0.81-1.20) | . | . | 27156 | . | 98 | . | 0.99 (0.81-1.20) |
| Specified indicator* | . | . | 9123 | . | 48 | . | 1.75 (1.29-2.38) | . | . | 7742 | . | 54 | . | 1.72 (1.29-2.29) | . | . | 2402 | . | 26 | . | 2.01 (1.35-3.00) | . | . | 1079 | . | 23 | . | 3.83 (2.50-5.87) | . | . | 274 | . | 7 | . | 5.98 (2.79-12.82) |
| Other indicator(s)^ | . | . | 7458 | . | 63 | . | 1.88 (1.45-2.43) | . | . | 8839 | . | 57 | . | 1.92 (1.46-2.53) | . | . | 14179 | . | 85 | . | 1.77 (1.39-2.24) | . | . | 15502 | . | 88 | . | 1.58 (1.26-1.98) | . | . | 16307 | . | 104 | . | 1.74 (1.41-2.14) |
|  | . | . |  | . |  | . | *p-het = <.0001* | . | . |  | . |  | . | *p-het = <.0001* | . | . |  | . |  | . | *p-het = <.0001* | . | . |  | . |  | . | *p-het = <.0001* | . | . |  | . |  | . | *p-het = <.0001* |
| **General anxiety disorder^d^** | | | | | | | | | | | | | | | | | | | | | | | | | | | | | | | | | | | |
| Low-risk drinkers | . | . | 25931 | . | 27 | . | 1.00 (0.67-1.48) | . | . | 25931 | . | 27 | . | 1.00 (0.67-1.48) | . | . | 25931 | . | 27 | . | 1.00 (0.67-1.48) | . | . | 25931 | . | 27 | . | 1.00 (0.67-1.48) | . | . | 25931 | . | 27 | . | 1.00 (0.67-1.48) |
| High-risk drinkers | . | . | 27220 | . | 34 | . | 0.96 (0.69-1.35) | . | . | 27220 | . | 34 | . | 0.96 (0.68-1.34) | . | . | 27220 | . | 34 | . | 0.96 (0.69-1.34) | . | . | 27220 | . | 34 | . | 0.96 (0.68-1.34) | . | . | 27220 | . | 34 | . | 0.96 (0.69-1.34) |
| Specified indicator* | . | . | 9149 | . | 22 | . | 1.83 (1.16-2.88) | . | . | 7774 | . | 22 | . | 1.73 (1.11-2.70) | . | . | 2416 | . | 12 | . | 2.60 (1.45-4.67) | . | . | 1093 | . | 9 | . | 4.03 (2.05-7.92) | . | . | 279 | . | 2 | . | 3.99 (0.98-16.29) |
| Other indicator(s)^ | . | . | 7494 | . | 27 | . | 2.11 (1.42-3.12) | . | . | 8869 | . | 27 | . | 2.22 (1.49-3.30) | . | . | 14227 | . | 37 | . | 1.81 (1.27-2.58) | . | . | 15550 | . | 40 | . | 1.75 (1.25-2.44) | . | . | 16364 | . | 47 | . | 1.94 (1.42-2.64) |
|  | . | . |  | . |  | . | *p-het = 0.0052* | . | . |  | . |  | . | *p-het = 0.0040* | . | . |  | . |  | . | *p-het = 0.0034* | . | . |  | . |  | . | *p-het = 0.0003* | . | . |  | . |  | . | *p-het = 0.0035* |
| **Panic attacks** | | | | | | | | | | | | | | | | | | | | | | | | | | | | | | | | | | | |
| Low-risk drinkers | . | . | 25805 | . | 153 | . | 1.00 (0.84-1.18) | . | . | 25805 | . | 153 | . | 1.00 (0.84-1.18) | . | . | 25805 | . | 153 | . | 1.00 (0.85-1.18) | . | . | 25805 | . | 153 | . | 1.00 (0.85-1.18) | . | . | 25805 | . | 153 | . | 1.00 (0.85-1.18) |
| High-risk drinkers | . | . | 27038 | . | 216 | . | 1.02 (0.89-1.17) | . | . | 27038 | . | 216 | . | 1.02 (0.89-1.16) | . | . | 27038 | . | 216 | . | 1.01 (0.89-1.16) | . | . | 27038 | . | 216 | . | 1.01 (0.89-1.16) | . | . | 27038 | . | 216 | . | 1.02 (0.89-1.16) |
| Specified indicator* | . | . | 9109 | . | 62 | . | 1.11 (0.86-1.44) | . | . | 7710 | . | 86 | . | 1.13 (0.90-1.41) | . | . | 2381 | . | 47 | . | 1.90 (1.41-2.56) | . | . | 1065 | . | 37 | . | 3.17 (2.26-4.45) | . | . | 267 | . | 14 | . | 5.90 (3.38-10.28) |
| Other indicator(s)^ | . | . | 7417 | . | 104 | . | 1.36 (1.12-1.66) | . | . | 8816 | . | 80 | . | 1.42 (1.13-1.78) | . | . | 14145 | . | 119 | . | 1.09 (0.90-1.32) | . | . | 15461 | . | 129 | . | 1.05 (0.88-1.27) | . | . | 16259 | . | 152 | . | 1.17 (0.99-1.39) |
|  | . | . |  | . |  | . | *p-het = 0.0804* | . | . |  | . |  | . | *p-het = 0.0604* | . | . |  | . |  | . | *p-het = 0.0015* | . | . |  | . |  | . | *p-het = <.0001* | . | . |  | . |  | . | *p-het = <.0001* |
| **Phobia** | | | | | | | | | | | | | | | | | | | | | | | | | | | | | | | | | | | |
| Low-risk drinkers | . | . | 25875 | . | 83 | . | 1.00 (0.80-1.26) | . | . | 25875 | . | 83 | . | 1.00 (0.80-1.26) | . | . | 25875 | . | 83 | . | 1.00 (0.80-1.25) | . | . | 25875 | . | 83 | . | 1.00 (0.80-1.26) | . | . | 25875 | . | 83 | . | 1.00 (0.80-1.26) |
| High-risk drinkers | . | . | 27159 | . | 95 | . | 0.95 (0.77-1.16) | . | . | 27159 | . | 95 | . | 0.94 (0.77-1.15) | . | . | 27159 | . | 95 | . | 0.94 (0.77-1.15) | . | . | 27159 | . | 95 | . | 0.95 (0.77-1.16) | . | . | 27159 | . | 95 | . | 0.95 (0.77-1.16) |
| Specified indicator* | . | . | 9139 | . | 32 | . | 1.09 (0.76-1.56) | . | . | 7760 | . | 36 | . | 0.93 (0.67-1.31) | . | . | 2402 | . | 26 | . | 1.79 (1.20-2.67) | . | . | 1089 | . | 13 | . | 1.88 (1.08-3.29) | . | . | 279 | . | 2 | . | 1.54 (0.38-6.26) |
| Other indicator(s)^ | . | . | 7467 | . | 54 | . | 1.34 (1.02-1.77) | . | . | 8846 | . | 50 | . | 1.58 (1.19-2.10) | . | . | 14204 | . | 60 | . | 1.07 (0.82-1.41) | . | . | 15517 | . | 73 | . | 1.16 (0.91-1.48) | . | . | 16327 | . | 84 | . | 1.23 (0.99-1.54) |
|  | . | . |  | . |  | . | *p-het = 0.2299* | . | . |  | . |  | . | *p-het = 0.0225* | . | . |  | . |  | . | *p-het = 0.0409* | . | . |  | . |  | . | *p-het = 0.1073* | . | . |  | . |  | . | *p-het = 0.3284* |

OR, odds ratio; CI, confidence interval; BMI, body mass index; CIDI-SF, composite international diagnostic interview short-form; HED, heavy episodic drinking

All models were adjusted for age group, region, education, income, marital status, prior chronic diseases, smoking and BMI.

Low-risk drinkers were current regular drinkers who drank <200g/week, with no HED in a typical drinking week or problem drinking indicator reported; high-risk drinkers were current regular drinkers who either drank at least 200g/week or engaged in HED in a typical drinking week, but with no problem drinking indicator reported.

*Reporting the problem drinking indicator specified in the column heading.

^Reporting any problem drinking indicator(s) except the indicator specified in the column heading.

^a^ Reporting one or more of the following for ≥ 3 days each week in the past month: delayed or fitful sleep; waking up too early; needing medicine to help sleep; having difficulty staying alert during daytime.

^b^ Reporting one or more of the following for ≥ 2 weeks in the past 12 months: feeling sad or depressed; loss of interest; loss of appetite; feeling worthless.

^c^ Assessed by the CIDI-SF (A).

^d^ Assessed by the CIDI-SF (B).

**Figure S7. Cross-sectional associations of problem drinking (excluding negative emotions indicator from the definition of problem drinking) with wellbeing in male current regular drinkers**

ORs were adjusted for age group, region, education, income, marital status, prior chronic diseases, smoking and BMI. Problem drinking indicators included: drinking in the morning; unable to work or do anything due to drinking; couldn't stop drinking; had shakes when stopping drinking. Each solid square represents an OR. 95% CIs are plotted using floating standard errors to allow for comparison between any two categories. The size of each box is inversely proportional to the ‘floated’ variance of the log OR in each group and the error bars indicate the group-specific 95% CI. OR, odds ratio; CI, confidence interval; BMI, body mass index; HED, heavy episodic drinking. Low-risk drinkers were current regular drinkers who drank <200g/week, with no HED in a typical drinking week or problem drinking indicator reported; high-risk drinkers were current regular drinkers who either drank at least 200g/week or engaged in HED in a typical drinking week, but with no problem drinking indicator reported; problem drinkers were current regular drinkers who reported at least one problem drinking indicator, and were further classified into “1 problem drinking indicator” and “2+ problem drinking indicators” according to the number of problem drinking indicators reported.

**Figure S8. Prospective associations of problem drinking with all-cause mortality, all hospitalisations and events due to all external causes in male current regular drinkers without prior chronic diseases, by rural and urban regions**

Models were stratified by age-at-risk and region, further adjusted for education, income, smoking, physical activity, fruit intake and BMI. Participants with prior coronary heart disease, stroke, transient ischaemic attack, diabetes, cancer, tuberculosis, chronic hepatitis/cirrhosis, rheumatoid arthritis, peptic ulcer, emphysema/bronchitis, gallstone/gallbladder disease or kidney disease were excluded from the analysis. Each solid square represents an HR. 95% CIs are plotted using floating standard errors to allow for comparison between any two categories. The size of each box is inversely proportional to the ‘floated’ variance of the log HR in each group and the error bars indicate the group-specific 95% CI. HR, hazard ratio; CI, confidence interval; BMI, body mass index; ICD-10, international classification of diseases version 10; HED, heavy episodic drinking. Low-risk drinkers were current regular drinkers who drank <200g/week, with no HED in a typical drinking week or problem drinking indicator reported; high-risk drinkers were current regular drinkers who either drank at least 200g/week or engaged in HED in a typical drinking week, but with no problem drinking indicator reported; problem drinkers were current regular drinkers who reported at least one problem drinking indicator, and were further classified into “1 problem drinking indicator” and “2+ problem drinking indicators” according to the number of problem drinking indicators reported.

**Table S9. Adjusted hazard ratios (HRs) associated with per an increase in the number of problem drinking indicators in men with at least one problem drinking indicator**

|  | | **Cases N** |  | **Non-cases N** |  |  | **Adjusted**  **HR (95% CI)** | ***p-value*** |  |
| --- | --- | --- | --- | --- | --- | --- | --- | --- | --- |
| **All-cause mortality** | . | 1401 | . | 12248 | . | . | 1.31 (1.20-1.43) | *<.0001* | . |
| **All hospitalisation** | . | 8017 | . | 5632 | . | . | 1.06 (1.01-1.10) | *0.0087* | . |
| **External causes (ICD-10:V01-Y98)^a^** | . | 153 | . | 13496 | . | . | 1.43 (1.15-1.78) | *0.0013* | . |

HR, hazard ratio; CI, confidence interval; BMI, body mass index; ICD-10, international classification of diseases version 10.

Models were stratified by age-at-risk and region, further adjusted for education, income, smoking, physical activity, fruit intake and BMI.

Participants with prior coronary heart disease, stroke, transient ischaemic attack, diabetes, cancer, tuberculosis, chronic hepatitis/cirrhosis, rheumatoid arthritis, peptic ulcer, emphysema/bronchitis, gallstone/gallbladder disease or kidney disease were excluded from the analysis.

^a^ Deaths and hospitalisations combined.

**Table S10. Prospective associations of problem drinking indicators with all-cause mortality, all hospitalisations, and events due to external causes in male current regular drinkers**

|  | | | **Morning drinking** | | | | |  | | **Unable to stop** | | | | |  | | **Unable to work** | | | | |  | | **Negative emotions** | | | | |  | | **Having shakes** | | | | | |
| --- | --- | --- | --- | --- | --- | --- | --- | --- | --- | --- | --- | --- | --- | --- | --- | --- | --- | --- | --- | --- | --- | --- | --- | --- | --- | --- | --- | --- | --- | --- | --- | --- | --- | --- | --- | --- |
|  | | | **Non- cases N** |  | **Cases N** |  | **HR (95% CI)** |  | | **Non- cases N** |  | **Cases N** |  | **HR (95% CI)** |  | | **Non- cases N** |  | **Cases N** |  | **HR (95% CI)** |  | | **Non- cases N** |  | **Cases N** |  | **HR (95% CI)** |  | | **Non- cases N** |  | **Cases N** |  | **HR (95% CI)** |  |
| **All-cause mortality** | | | | | | | | | | | | | | | | | | | | | | | | | | | | | | | | | | | | |
| Low-risk drinkers | . | . | 19368 | . | 1277 | . | 1.00 (0.94-1.06) | . | . | 19368 | . | 1277 | . | 1.00 (0.94-1.06) | . | . | 19368 | . | 1277 | . | 1.00 (0.94-1.06) | . | . | 19368 | . | 1277 | . | 1.00 (0.94-1.06) | . | . | 19368 | . | 1277 | . | 1.00 (0.94-1.06) |  |
| High-risk drinkers | . | . | 21262 | . | 1610 | . | 1.26 (1.20-1.33) | . | . | 21262 | . | 1610 | . | 1.27 (1.21-1.34) | . | . | 21262 | . | 1610 | . | 1.27 (1.21-1.34) | . | . | 21262 | . | 1610 | . | 1.27 (1.21-1.34) | . | . | 21262 | . | 1610 | . | 1.27 (1.21-1.34) |  |
| Specified indicator* | . | . | 6642 | . | 986 | . | 1.68 (1.57-1.80) | . | . | 5635 | . | 622 | . | 1.42 (1.31-1.55) | . | . | 1880 | . | 122 | . | 1.41 (1.18-1.69) | . | . | 806 | . | 60 | . | 1.59 (1.23-2.05) | . | . | 187 | . | 41 | . | 3.17 (2.33-4.31) |  |
| Other indicator(s)^ | . | . | 5606 | . | 415 | . | 1.13 (1.02-1.24) | . | . | 6613 | . | 779 | . | 1.50 (1.39-1.61) | . | . | 10368 | . | 1279 | . | 1.47 (1.39-1.56) | . | . | 11442 | . | 1341 | . | 1.46 (1.38-1.54) | . | . | 12061 | . | 1360 | . | 1.44 (1.36-1.52) |  |
|  | . | . |  | . |  | . | *p-het = <.0001* | . | . |  | . |  | . | *p-het = <.0001* | . | . |  | . |  | . | *p-het = <.0001* | . | . |  | . |  | . | *p-het = <.0001* | . | . |  | . |  | . | *p-het = <.0001* |  |
| **All hospitalisations** | | | | | | | | | | | | | | | | | | | | | | | | | | | | | | | | | | | | |
| Low-risk drinkers | . | . | 10664 | . | 9981 | . | 1.00 (0.98-1.02) | . | . | 10664 | . | 9981 | . | 1.00 (0.98-1.02) | . | . | 10664 | . | 9981 | . | 1.00 (0.98-1.02) | . | . | 10664 | . | 9981 | . | 1.00 (0.98-1.02) | . | . | 10664 | . | 9981 | . | 1.00 (0.98-1.02) |  |
| High-risk drinkers | . | . | 11566 | . | 11306 | . | 1.09 (1.07-1.11) | . | . | 11566 | . | 11306 | . | 1.09 (1.07-1.11) | . | . | 11566 | . | 11306 | . | 1.09 (1.07-1.11) | . | . | 11566 | . | 11306 | . | 1.09 (1.07-1.11) | . | . | 11566 | . | 11306 | . | 1.09 (1.07-1.11) |  |
| Specified indicator* | . | . | 2717 | . | 4911 | . | 1.09 (1.06-1.12) | . | . | 2635 | . | 3622 | . | 1.12 (1.08-1.16) | . | . | 1081 | . | 921 | . | 1.11 (1.04-1.19) | . | . | 475 | . | 391 | . | 1.03 (0.93-1.14) | . | . | 99 | . | 129 | . | 1.29 (1.08-1.53) |  |
| Other indicator(s)^ | . | . | 2915 | . | 3106 | . | 1.08 (1.05-1.12) | . | . | 2997 | . | 4395 | . | 1.06 (1.03-1.10) | . | . | 4551 | . | 7096 | . | 1.08 (1.06-1.11) | . | . | 5157 | . | 7626 | . | 1.09 (1.06-1.12) | . | . | 5533 | . | 7888 | . | 1.08 (1.06-1.11) |  |
|  | . | . |  | . |  | . | *p-het = <.0001* | . | . |  | . |  | . | *p-het = <.0001* | . | . |  | . |  | . | *p-het = <.0001* | . | . |  | . |  | . | *p-het = <.0001* | . | . |  | . |  | . | *p-het = <.0001* |  |
| **External causes (ICD-10:V01-Y98)^a^** | | | | | | | | | | | | | | | | | | | | | | | | | | | | | | | | | | | | |
| Low-risk drinkers | . | . | 20530 | . | 115 | . | 1.00 (0.83-1.21) | . | . | 20530 | . | 115 | . | 1.00 (0.83-1.21) | . | . | 20530 | . | 115 | . | 1.00 (0.83-1.21) | . | . | 20530 | . | 115 | . | 1.00 (0.83-1.21) | . | . | 20530 | . | 115 | . | 1.00 (0.83-1.21) |  |
| High-risk drinkers | . | . | 22708 | . | 164 | . | 1.15 (0.98-1.34) | . | . | 22708 | . | 164 | . | 1.16 (0.99-1.36) | . | . | 22708 | . | 164 | . | 1.16 (0.99-1.35) | . | . | 22708 | . | 164 | . | 1.16 (0.99-1.35) | . | . | 22708 | . | 164 | . | 1.16 (0.99-1.35) |  |
| Specified indicator* | . | . | 7518 | . | 110 | . | 1.63 (1.33-2.00) | . | . | 6184 | . | 73 | . | 1.50 (1.18-1.91) | . | . | 1988 | . | 14 | . | 0.97 (0.57-1.66) | . | . | 854 | . | 12 | . | 1.96 (1.10-3.47) | . | . | 222 | . | 6 | . | 3.52 (1.57-7.87) |  |
| Other indicator(s)^ | . | . | 5978 | . | 43 | . | 0.97 (0.71-1.32) | . | . | 7312 | . | 80 | . | 1.27 (1.01-1.59) | . | . | 11508 | . | 139 | . | 1.43 (1.20-1.71) | . | . | 12642 | . | 141 | . | 1.33 (1.11-1.58) | . | . | 13274 | . | 147 | . | 1.33 (1.12-1.57) |  |
|  | . | . |  | . |  | . | *p-het = 0.0024* | . | . |  | . |  | . | *p-het = 0.0710* | . | . |  | . |  | . | *p-het = 0.0476* | . | . |  | . |  | . | *p-het = 0.0537* | . | . |  | . |  | . | *p-het = 0.0077* |  |

HR, hazard ratio; CI, confidence interval; BMI, body mass index; ICD-10, international classification of diseases version 10; HED, heavy episodic drinking.

Models were stratified by age-at-risk and region, further adjusted for education, income, smoking, physical activity, fruit intake and BMI.

Participants with prior coronary heart disease, stroke, transient ischaemic attack, diabetes, cancer, tuberculosis, chronic hepatitis/cirrhosis, rheumatoid arthritis, peptic ulcer, emphysema/bronchitis, gallstone/gallbladder disease or kidney disease were excluded from the analysis.

Low-risk drinkers were current regular drinkers who drank <200g/week, with no HED in a typical drinking week or problem drinking indicator reported; high-risk drinkers were current regular drinkers who either drank at least 200g/week or engaged in HED in a typical drinking week, but with no problem drinking indicator reported.

*Reporting the problem drinking indicator specified in the column heading.

^Reporting any problem drinking indicator(s) except the indicator specified in the column heading.

^a^ Deaths and hospitalisations combined.

**Table S11. Cross-sectional associations of stressful life events with problem drinking in male current regular drinkers in sequentially adjusted models**

|  | | | | | | | | | | **Age & Region** | |  | | **+Education & Income** | |  | | **Main model (+Smoking)** | |  | | **+Prior chronic diseases** | |  | | **+Prior psychiatric disorders** | | |  |
| --- | --- | --- | --- | --- | --- | --- | --- | --- | --- | --- | --- | --- | --- | --- | --- | --- | --- | --- | --- | --- | --- | --- | --- | --- | --- | --- | --- | --- | --- |
|  | | | | **Total regular drinkers**  **N** |  | | **Problem drinkers^a^**  **N (%)** |  | | **OR (95% CI)** | ***p-value*** |  | | **OR (95% CI)** | ***p-value*** |  | | **OR (95% CI)** | ***p-value*** |  | | **OR (95% CI)** | ***p-value*** |  | | **OR (95% CI)** | ***p-value*** | |  |
| **Family-related events** | | | | | | | | | | | | | | | | | | | | | | | | | | | | | |
| Divorce or separation | | | | | | | | | | | | | | | | | | | | | | | | | | | | | |
| No | . | . | . | 69701 | . | . | 16639 (23.9%) | . | . | 1.00 |  | . | . | 1.00 |  | . | . | 1.00 |  | . | . | 1.00 |  | . | . | 1.00 |  |  |  |
| Yes | . | . | . | 203 | . | . | 53 (26.1%) | . | . | 1.45 (1.04-2.01) | *0.0274* | . | . | 1.38 (0.99-1.92) | *0.0558* | . | . | 1.32 (0.95-1.83) | *0.1027* | . | . | 1.31 (0.94-1.83) | *0.1059* | . | . | 1.31 (0.94-1.82) | *0.1088* |  |  |
| Family conflict | | | | | | | | | | | | | | | | | | | | | | | | | | | | | |
| No | . | . | . | 69417 | . | . | 16540 (23.8%) | . | . | 1.00 |  | . | . | 1.00 |  | . | . | 1.00 |  | . | . | 1.00 |  | . | . | 1.00 |  |  |  |
| Yes | . | . | . | 487 | . | . | 152 (31.2%) | . | . | 1.52 (1.24-1.87) | *<.0001* | . | . | 1.51 (1.23-1.86) | *<.0001* | . | . | 1.50 (1.22-1.84) | *0.0001* | . | . | 1.49 (1.21-1.83) | *0.0001* | . | . | 1.49 (1.21-1.82) | *0.0002* |  |  |
| Death of spouse | | | | | | | | | | | | | | | | | | | | | | | | | | | | | |
| No | . | . | . | 69550 | . | . | 16596 (23.8%) | . | . | 1.00 |  | . | . | 1.00 |  | . | . | 1.00 |  | . | . | 1.00 |  | . | . | 1.00 |  |  |  |
| Yes | . | . | . | 327 | . | . | 96 (29.4%) | . | . | 1.11 (0.87-1.42) | *0.3881* | . | . | 1.07 (0.83-1.36) | *0.6033* | . | . | 1.07 (0.83-1.36) | *0.6133* | . | . | 1.07 (0.83-1.36) | *0.6169* | . | . | 1.06 (0.83-1.36) | *0.6202* |  |  |
| Death or major illness of other family member | | | | | | | | | | | | | | | | | | | | | | | | | | | | | |
| No | . | . | . | 66680 | . | . | 15871 (23.8%) | . | . | 1.00 |  | . | . | 1.00 |  | . | . | 1.00 |  | . | . | 1.00 |  | . | . | 1.00 |  |  |  |
| Yes | . | . | . | 3224 | . | . | 821 (25.5%) | . | . | 1.22 (1.12-1.33) | *<.0001* | . | . | 1.20 (1.10-1.31) | *<.0001* | . | . | 1.20 (1.10-1.30) | *<.0001* | . | . | 1.19 (1.09-1.30) | *<.0001* | . | . | 1.19 (1.09-1.30) | *<.0001* |  |  |
| \| Any family-related events \| \| \| \| \| \| \| \| \| \| \| \| \| \| \| \| \| \| \| \| \| \| \| \| \| \| \| \| \| \| --- \| --- \| --- \| --- \| --- \| --- \| --- \| --- \| --- \| --- \| --- \| --- \| --- \| --- \| --- \| --- \| --- \| --- \| --- \| --- \| --- \| --- \| --- \| --- \| --- \| --- \| --- \| --- \| --- \| \| No \| . \| . \| . \| 65748 \| . \| . \| 15606 (23.7%) \| . \| . \| 1.00 \|  \| . \| . \| 1.00 \|  \| . \| . \| 1.00 \|  \| . \| . \| 1.00 \|  \| . \| . \| 1.00 \|  \| \| Yes \| . \| . \| . \| 4156 \| . \| . \| 1086 (26.1%) \| . \| . \| 1.24 (1.15-1.34) \| *<.0001* \| . \| . \| 1.22 (1.13-1.32) \| *<.0001* \| . \| . \| 1.21 (1.13-1.31) \| *<.0001* \| . \| . \| 1.21 (1.12-1.31) \| *<.0001* \| . \| . \| 1.21 (1.12-1.31) \| *<.0001* \| | | | | | | | | | | | | | | | | | | | | | | | | | | | | | |
| **Finance-related events** | | | | | | | | | | | | | | | | | | | | | | | | | | | | | |
| Job loss or retirement | | | | | | | | | | | | | | | | | | | | | | | | | | | | | |
| No | . | . | . | 69587 | . | . | 16622 (23.8%) | . | . | 1.00 |  | . | . | 1.00 |  | . | . | 1.00 |  | . | . | 1.00 |  | . | . | 1.00 |  |  |  |
| Yes | . | . | . | 317 | . | . | 70 (22.1%) | . | . | 1.59 (1.20-2.10) | *0.0012* | . | . | 1.44 (1.08-1.90) | *0.0115* | . | . | 1.45 (1.09-1.91) | *0.0102* | . | . | 1.44 (1.09-1.91) | *0.0110* | . | . | 1.44 (1.09-1.90) | *0.0113* |  |  |
| Bankruptcy | | | | | | | | | | | | | | | | | | | | | | | | | | | | | |
| No | . | . | . | 69683 | . | . | 16608 (23.8%) | . | . | 1.00 |  | . | . | 1.00 |  | . | . | 1.00 |  | . | . | 1.00 |  | . | . | 1.00 |  |  |  |
| Yes | . | . | . | 221 | . | . | 84 (38.0%) | . | . | 1.51 (1.14-2.00) | *0.0041* | . | . | 1.47 (1.10-1.94) | *0.0080* | . | . | 1.48 (1.11-1.96) | *0.0068* | . | . | 1.47 (1.11-1.96) | *0.0075* | . | . | 1.47 (1.11-1.96) | *0.0074* |  |  |
| Loss of income or debt | | | | | | | | | | | | | | | | | | | | | | | | | | | | | |
| No | . | . | . | 69592 | . | . | 16577 (23.8%) | . | . | 1.00 |  | . | . | 1.00 |  | . | . | 1.00 |  | . | . | 1.00 |  | . | . | 1.00 |  |  |  |
| Yes | . | . | . | 312 | . | . | 115 (36.9%) | . | . | 2.10 (1.63-2.69) | *<.0001* | . | . | 1.85 (1.44-2.37) | *<.0001* | . | . | 1.86 (1.45-2.39) | *<.0001* | . | . | 1.85 (1.44-2.38) | *<.0001* | . | . | 1.85 (1.44-2.37) | *<.0001* |  |  |
| Any finance-related events | | | | | | | | | | | | | | | | | | | | | | | | | | | | | |
| No | . | . | . | 69099 | . | . | 16441 (23.8%) | . | . | 1.00 |  | . | . | 1.00 |  | . | . | 1.00 |  | . | . | 1.00 |  | . | . | 1.00 |  |  |  |
| Yes | . | . | . | 805 | . | . | 251 (31.1%) | . | . | 1.69 (1.44-1.99) | *<.0001* | . | . | 1.56 (1.33-1.83) | *<.0001* | . | . | 1.57 (1.34-1.84) | *<.0001* | . | . | 1.56 (1.33-1.84) | *<.0001* | . | . | 1.56 (1.33-1.83) | *<.0001* |  |  |
| **Injury-related events** | | | | | | | | | | | | | | | | | | | | | | | | | | | | | |
| Violence | | | | | | | | | | | | | | | | | | | | | | | | | | | | | |
| No | . | . | . | 69796 | . | . | 16653 (23.9%) | . | . | 1.00 |  | . | . | 1.00 |  | . | . | 1.00 |  | . | . | 1.00 |  | . | . | 1.00 |  |  |  |
| Yes | . | . | . | 108 | . | . | 39 (36.1%) | . | . | 1.68 (1.11-2.55) | *0.0143* | . | . | 1.67 (1.10-2.54) | *0.0155* | . | . | 1.68 (1.11-2.56) | *0.0149* | . | . | 1.68 (1.10-2.55) | *0.0156* | . | . | 1.67 (1.10-2.54) | *0.0161* |  |  |
| Major injury or traffic accident | | | | | | | | | | | | | | | | | | | | | | | | | | | | | |
| No | . | . | . | 69403 | . | . | 16566 (23.9%) | . | . | 1.00 |  | . | . | 1.00 |  | . | . | 1.00 |  | . | . | 1.00 |  | . | . | 1.00 |  |  |  |
| Yes | . | . | . | 501 | . | . | 126 (25.1%) | . | . | 1.18 (0.95-1.46) | *0.1355* | . | . | 1.16 (0.94-1.44) | *0.1663* | . | . | 1.15 (0.93-1.43) | *0.1929* | . | . | 1.15 (0.93-1.43) | *0.2001* | . | . | 1.15 (0.93-1.43) | *0.2040* |  |  |
| Natural disaster | | | | | | | | | | | | | | | | | | | | | | | | | | | | | |
| No | . | . | . | 69848 | . | . | 16679 (23.9%) | . | . | 1.00 |  | . | . | 1.00 |  | . | . | 1.00 |  | . | . | 1.00 |  | . | . | 1.00 |  |  |  |
| Yes | . | . | . | 56 | . | . | 13 (23.2%) | . | . | 1.09 (0.56-2.09) | *0.8057* | . | . | 1.05 (0.54-2.02) | *0.8862* | . | . | 1.05 (0.54-2.02) | *0.8869* | . | . | 1.05 (0.54-2.03) | *0.8819* | . | . | 1.05 (0.54-2.03) | *0.8803* |  |  |
| Any injury or disaster events | | | | | | | | | | | | | | | | | | | | | | | | | | | | | |
| No | . | . | . | 69244 | . | . | 16516 (23.9%) | . | . | 1.00 |  | . | . | 1.00 |  | . | . | 1.00 |  | . | . | 1.00 |  | . | . | 1.00 |  |  |  |
| Yes | . | . | . | 660 | . | . | 176 (26.7%) | . | . | 1.24 (1.03-1.49) | *0.0210* | . | . | 1.23 (1.02-1.48) | *0.0297* | . | . | 1.22 (1.01-1.47) | *0.0347* | . | . | 1.22 (1.01-1.47) | *0.0367* | . | . | 1.22 (1.01-1.46) | *0.0380* |  |  |
| **Any major stressful life event** | | | | | | | | | | | | | | | | | | | | | | | | | | | | | |
| No | . | . | . | 64509 | . | . | 15266 (23.7%) | . | . | 1.00 |  | . | . | 1.00 |  | . | . | 1.00 |  | . | . | 1.00 |  | . | . | 1.00 |  |  |  |
| Yes | . | . | . | 5395 | . | . | 1426 (26.4%) | . | . | 1.28 (1.20-1.37) | *<.0001* | . | . | 1.25 (1.17-1.33) | *<.0001* | . | . | 1.24 (1.16-1.33) | *<.0001* | . | . | 1.24 (1.16-1.33) | *<.0001* | . | . | 1.24 (1.16-1.33) | *<.0001* |  |  |

OR, odds ratio; CI, confidence interval.

All p-values are for Wald chi-squared test.

^a^ Reporting one or more in the past month of: drinking in the morning, unable to work or do anything due to drinking; depressed irritated or lost control due to drinking; couldn't stop drinking; having shakes when stopping drinking.

**Table S12. Cross-sectional associations of problem drinking with wellbeing-related measures in male current regular drinkers in sequentially adjusted models**

|  | | | | | | | | | **Age & Region** | |  | | **+Education & Income** | |  | | **Main model (+Marital status, prior chronic disease, smoking, BMI)** | | |  | | **+Prior psychiatric disorder** | |  | | **+Physical activity** | |
| --- | --- | --- | --- | --- | --- | --- | --- | --- | --- | --- | --- | --- | --- | --- | --- | --- | --- | --- | --- | --- | --- | --- | --- | --- | --- | --- | --- |
|  | | | **Cases N** |  | **Non-cases N** |  | | | **OR (95% CI)** | ***p-het*** |  | | **OR (95% CI)** | ***p-het*** |  | | **OR (95% CI)** | | ***p-het*** |  | | **OR (95% CI)** | ***p-het*** |  | | **OR (95% CI)** | ***p-het*** |
| **Poor self-reported health** | | | | | | | | | | | | | | | | | | | | | | | | | | | |
| Low-risk drinkers | . | . | 1480 | . | 24478 | . | . | . | 1.00 (0.95-1.06) | *.* | . | . | 1.00 (0.95-1.06) | *.* | . | . | | 1.00 (0.94-1.06) | *.* | . | . | 1.00 (0.94-1.06) | *.* | . | . | 1.00 (0.94-1.06) | *.* |
| High-risk drinkers | . | . | 1603 | . | 25651 | . | . | . | 0.99 (0.94-1.04) | *.* | . | . | 0.97 (0.92-1.02) | *.* | . | . | | 0.97 (0.92-1.02) | *.* | . | . | 0.97 (0.92-1.02) | *.* | . | . | 0.97 (0.92-1.02) | *.* |
| 1 problem drinking indicator | . | . | 951 | . | 12320 | . | . | . | 1.18 (1.10-1.26) | *.* | . | . | 1.12 (1.04-1.20) | *.* | . | . | | 1.09 (1.02-1.17) | *.* | . | . | 1.09 (1.02-1.17) | *.* | . | . | 1.09 (1.02-1.17) | *.* |
| 2+ problem drinking indicators | . | . | 362 | . | 3059 | . | . | . | 1.70 (1.52-1.91) | *<.0001* | . | . | 1.60 (1.43-1.79) | *<.0001* | . | . | | 1.54 (1.37-1.73) | *<.0001* | . | . | 1.54 (1.37-1.73) | *<.0001* | . | . | 1.54 (1.37-1.72) | *<.0001* |
| **Life dissatisfaction** | | | | | | | | | | | | | | | | | | | | | | | | | | | |
| Low-risk drinkers | . | . | 1137 | . | 24821 | . | . | . | 1.00 (0.94-1.06) | *.* | . | . | 1.00 (0.94-1.06) | *.* | . | . | | 1.00 (0.94-1.07) | *.* | . | . | 1.00 (0.94-1.07) | *.* | . | . | 1.00 (0.94-1.07) | *.* |
| High-risk drinkers | . | . | 1136 | . | 26118 | . | . | . | 1.04 (0.98-1.10) | *.* | . | . | 1.02 (0.96-1.08) | *.* | . | . | | 1.00 (0.94-1.07) | *.* | . | . | 1.00 (0.94-1.07) | *.* | . | . | 1.00 (0.94-1.07) | *.* |
| 1 problem drinking indicator | . | . | 555 | . | 12716 | . | . | . | 1.40 (1.28-1.53) | *.* | . | . | 1.24 (1.14-1.36) | *.* | . | . | | 1.21 (1.11-1.33) | *.* | . | . | 1.21 (1.11-1.33) | *.* | . | . | 1.21 (1.10-1.33) | *.* |
| 2+ problem drinking indicators | . | . | 193 | . | 3228 | . | . | . | 1.99 (1.71-2.31) | *<.0001* | . | . | 1.70 (1.45-1.98) | *<.0001* | . | . | | 1.62 (1.39-1.89) | *<.0001* | . | . | 1.62 (1.39-1.89) | *<.0001* | . | . | 1.62 (1.39-1.89) | *<.0001* |
| **Sleep problem^a^** | | | | | | | | | | | | | | | | | | | | | | | | | | | |
| Low-risk drinkers | . | . | 3155 | . | 22803 | . | . | . | 1.00 (0.96-1.04) | *.* | . | . | 1.00 (0.96-1.04) | *.* | . | . | | 1.00 (0.96-1.04) | *.* | . | . | 1.00 (0.96-1.04) | *.* | . | . | 1.00 (0.96-1.04) | *.* |
| High-risk drinkers | . | . | 3503 | . | 23751 | . | . | . | 1.07 (1.03-1.11) | *.* | . | . | 1.06 (1.02-1.10) | *.* | . | . | | 1.08 (1.04-1.12) | *.* | . | . | 1.08 (1.04-1.12) | *.* | . | . | 1.08 (1.04-1.12) | *.* |
| 1 problem drinking indicator | . | . | 2313 | . | 10958 | . | . | . | 1.34 (1.28-1.41) | *.* | . | . | 1.31 (1.25-1.37) | *.* | . | . | | 1.31 (1.25-1.37) | *.* | . | . | 1.31 (1.25-1.37) | *.* | . | . | 1.31 (1.25-1.37) | *.* |
| 2+ problem drinking indicators | . | . | 652 | . | 2769 | . | . | . | 1.48 (1.36-1.61) | *<.0001* | . | . | 1.43 (1.31-1.56) | *<.0001* | . | . | | 1.42 (1.30-1.55) | *<.0001* | . | . | 1.42 (1.30-1.55) | *<.0001* | . | . | 1.42 (1.30-1.55) | *<.0001* |
| **Depression symptoms^b^** | | | | | | | | | | | | | | | | | | | | | | | | | | | |
| Low-risk drinkers | . | . | 462 | . | 25496 | . | . | . | 1.00 (0.91-1.10) | *.* | . | . | 1.00 (0.91-1.10) | *.* | . | . | | 1.00 (0.91-1.10) | *.* | . | . | 1.00 (0.91-1.10) | *.* | . | . | 1.00 (0.91-1.10) | *.* |
| High-risk drinkers | . | . | 480 | . | 26774 | . | . | . | 0.95 (0.87-1.04) | *.* | . | . | 0.96 (0.87-1.05) | *.* | . | . | | 0.95 (0.87-1.04) | *.* | . | . | 0.95 (0.87-1.04) | *.* | . | . | 0.95 (0.86-1.04) | *.* |
| 1 problem drinking indicator | . | . | 400 | . | 12871 | . | . | . | 1.57 (1.42-1.75) | *.* | . | . | 1.54 (1.39-1.70) | *.* | . | . | | 1.49 (1.34-1.65) | *.* | . | . | 1.48 (1.34-1.64) | *.* | . | . | 1.48 (1.33-1.64) | *.* |
| 2+ problem drinking indicators | . | . | 171 | . | 3250 | . | . | . | 2.53 (2.16-2.96) | *<.0001* | . | . | 2.45 (2.10-2.87) | *<.0001* | . | . | | 2.30 (1.97-2.70) | *<.0001* | . | . | 2.29 (1.96-2.69) | *<.0001* | . | . | 2.29 (1.95-2.68) | *<.0001* |
| **Major depressive episode^c^** | | | | | | | | | | | | | | | | | | | | | | | | | | | |
| Low-risk drinkers | . | . | 93 | . | 25865 | . | . | . | 1.00 (0.81-1.23) | *.* | . | . | 1.00 (0.81-1.23) | *.* | . | . | | 1.00 (0.81-1.24) | *.* | . | . | 1.00 (0.81-1.24) | *.* | . | . | 1.00 (0.81-1.24) | *.* |
| High-risk drinkers | . | . | 89 | . | 27165 | . | . | . | 0.91 (0.74-1.13) | *.* | . | . | 0.91 (0.74-1.12) | *.* | . | . | | 0.90 (0.73-1.11) | *.* | . | . | 0.90 (0.72-1.11) | *.* | . | . | 0.89 (0.72-1.10) | *.* |
| 1 problem drinking indicator | . | . | 66 | . | 13205 | . | . | . | 1.26 (0.99-1.62) | *.* | . | . | 1.20 (0.93-1.54) | *.* | . | . | | 1.15 (0.90-1.48) | *.* | . | . | 1.13 (0.88-1.45) | *.* | . | . | 1.12 (0.87-1.44) | *.* |
| 2+ problem drinking indicators | . | . | 25 | . | 3396 | . | . | . | 1.83 (1.23-2.72) | *0.0104* | . | . | 1.72 (1.16-2.57) | *0.0296* | . | . | | 1.60 (1.07-2.39) | *0.0700* | . | . | 1.55 (1.04-2.32) | *0.1015* | . | . | 1.54 (1.03-2.31) | *0.1023* |
| **Anxiety symptom** | | | | | | | | | | | | | | | | | | | | | | | | | | | |
| Low-risk drinkers | . | . | 75 | . | 25883 | . | . | . | 1.00 (0.79-1.27) | *.* | . | . | 1.00 (0.79-1.27) | *.* | . | . | | 1.00 (0.79-1.27) | *.* | . | . | 1.00 (0.79-1.27) | *.* | . | . | 1.00 (0.79-1.27) | *.* |
| High-risk drinkers | . | . | 98 | . | 27156 | . | . | . | 0.99 (0.81-1.21) | *.* | . | . | 0.99 (0.81-1.21) | *.* | . | . | | 0.99 (0.81-1.21) | *.* | . | . | 0.99 (0.81-1.21) | *.* | . | . | 0.99 (0.81-1.21) | *.* |
| 1 problem drinking indicator | . | . | 77 | . | 13194 | . | . | . | 1.74 (1.38-2.19) | *.* | . | . | 1.69 (1.34-2.13) | *.* | . | . | | 1.66 (1.32-2.10) | *.* | . | . | 1.65 (1.31-2.08) | *.* | . | . | 1.65 (1.30-2.08) | *.* |
| 2+ problem drinking indicators | . | . | 34 | . | 3387 | . | . | . | 2.48 (1.76-3.50) | *<.0001* | . | . | 2.42 (1.71-3.41) | *<.0001* | . | . | | 2.36 (1.67-3.34) | *<.0001* | . | . | 2.34 (1.66-3.31) | *<.0001* | . | . | 2.33 (1.65-3.29) | *<.0001* |
| **General anxiety disorder^d^** | | | | | | | | | | | | | | | | | | | | | | | | | | | |
| Low-risk drinkers | . | . | 27 | . | 25931 | . | . | . | 1.00 (0.67-1.48) | *.* | . | . | 1.00 (0.67-1.48) | *.* | . | . | | 1.00 (0.67-1.49) | *.* | . | . | 1.00 (0.67-1.49) | *.* | . | . | 1.00 (0.67-1.49) | *.* |
| High-risk drinkers | . | . | 34 | . | 27220 | . | . | . | 0.94 (0.67-1.32) | *.* | . | . | 0.96 (0.68-1.35) | *.* | . | . | | 0.97 (0.69-1.36) | *.* | . | . | 0.96 (0.68-1.35) | *.* | . | . | 0.96 (0.68-1.35) | *.* |
| 1 problem drinking indicator | . | . | 33 | . | 13238 | . | . | . | 1.81 (1.27-2.57) | *.* | . | . | 1.78 (1.25-2.53) | *.* | . | . | | 1.74 (1.22-2.47) | *.* | . | . | 1.72 (1.21-2.45) | *.* | . | . | 1.72 (1.21-2.45) | *.* |
| 2+ problem drinking indicators | . | . | 16 | . | 3405 | . | . | . | 2.86 (1.74-4.71) | *0.0005* | . | . | 2.85 (1.73-4.69) | *0.0007* | . | . | | 2.82 (1.71-4.66) | *0.0010* | . | . | 2.79 (1.69-4.61) | *0.0012* | . | . | 2.79 (1.69-4.61) | *0.0012* |
| **Panic attacks** | | | | | | | | | | | | | | | | | | | | | | | | | | | |
| Low-risk drinkers | . | . | 153 | . | 25805 | . | . | . | 1.00 (0.85-1.18) | *.* | . | . | 1.00 (0.85-1.18) | *.* | . | . | | 1.00 (0.84-1.18) | *.* | . | . | 1.00 (0.84-1.18) | *.* | . | . | 1.00 (0.84-1.18) | *.* |
| High-risk drinkers | . | . | 216 | . | 27038 | . | . | . | 0.99 (0.87-1.13) | *.* | . | . | 1.00 (0.87-1.14) | *.* | . | . | | 1.02 (0.90-1.17) | *.* | . | . | 1.02 (0.89-1.17) | *.* | . | . | 1.02 (0.89-1.16) | *.* |
| 1 problem drinking indicator | . | . | 105 | . | 13166 | . | . | . | 1.04 (0.85-1.26) | *.* | . | . | 1.03 (0.84-1.26) | *.* | . | . | | 1.05 (0.87-1.29) | *.* | . | . | 1.05 (0.86-1.28) | *.* | . | . | 1.05 (0.86-1.28) | *.* |
| 2+ problem drinking indicators | . | . | 61 | . | 3360 | . | . | . | 1.86 (1.44-2.41) | *0.0002* | . | . | 1.85 (1.42-2.39) | *0.0003* | . | . | | 1.92 (1.48-2.50) | *0.0002* | . | . | 1.92 (1.48-2.50) | *0.0001* | . | . | 1.92 (1.48-2.49) | *0.0002* |
| **Phobia** | | | | | | | | | | | | | | | | | | | | | | | | | | | |
| Low-risk drinkers | . | . | 83 | . | 25875 | . | . | . | 1.00 (0.80-1.25) | *.* | . | . | 1.00 (0.80-1.25) | *.* | . | . | | 1.00 (0.80-1.26) | *.* | . | . | 1.00 (0.80-1.26) | *.* | . | . | 1.00 (0.80-1.26) | *.* |
| High-risk drinkers | . | . | 95 | . | 27159 | . | . | . | 0.91 (0.75-1.12) | *.* | . | . | 0.91 (0.74-1.11) | *.* | . | . | | 0.95 (0.77-1.16) | *.* | . | . | 0.94 (0.77-1.15) | *.* | . | . | 0.94 (0.77-1.16) | *.* |
| 1 problem drinking indicator | . | . | 68 | . | 13203 | . | . | . | 1.31 (1.03-1.67) | *.* | . | . | 1.26 (0.99-1.61) | *.* | . | . | | 1.29 (1.01-1.65) | *.* | . | . | 1.28 (1.00-1.63) | *.* | . | . | 1.28 (1.00-1.64) | *.* |
| 2+ problem drinking indicators | . | . | 18 | . | 3403 | . | . | . | 1.10 (0.69-1.75) | *0.1618* | . | . | 1.05 (0.66-1.68) | *0.2438* | . | . | | 1.08 (0.68-1.72) | *0.2752* | . | . | 1.07 (0.67-1.71) | *0.2914* | . | . | 1.08 (0.68-1.72) | *0.2846* |

OR, odds ratio; CI, confidence interval; BMI, body mass index; CIDI-SF, composite international diagnostic interview short-form; HED, heavy episodic drinking.

Low-risk drinkers were current regular drinkers who drank <200g/week, with no HED in a typical drinking week or problem drinking indicator reported; high-risk drinkers were current regular drinkers who either drank at least 200g/week or engaged in HED in a typical drinking week, but with no problem drinking indicator reported; problem drinkers were current regular drinkers who reported at least one problem drinking indicator, and were further classified into “1 problem drinking indicator” and “2+ problem drinking indicators” according to the number of problem drinking indicators reported.

^a^ Reporting one or more of the following for >= 3 days each week in the past month: delayed or fitful sleep; waking up too early; needing medicine to help sleep; having difficulty staying alert during daytime.

^b^ Reporting one or more of the following for >=2 weeks in the past 12 months: feeling sad or depressed; loss of interest; loss of appetite; feeling worthless.

^c^ Assessed by the CIDI-SF (A).

^d^ Assessed by the CIDI-SF (B).

**Table S13. Prospective associations of problem drinking with all-cause mortality, all hospitalisations, and events due to external causes in male current regular drinkers in sequentially adjusted models**

|  | | | | | | | | **Age & Region** | | | | | |  | | | **+Education & Income** | | | | |  | | **+Smoking status** | | | | | |  | | **+BMI** | | | | | |  | | **Main model (+Fruit intake & Physical activity)** | | | | | |  | | **+Prior psychiatric diseases** | | | | | |  |  |  |
| --- | --- | --- | --- | --- | --- | --- | --- | --- | --- | --- | --- | --- | --- | --- | --- | --- | --- | --- | --- | --- | --- | --- | --- | --- | --- | --- | --- | --- | --- | --- | --- | --- | --- | --- | --- | --- | --- | --- | --- | --- | --- | --- | --- | --- | --- | --- | --- | --- | --- | --- | --- | --- | --- | --- | --- | --- |
|  | **Non-cases N** | |  | **Cases N** | |  | | | | **HR (95% CI)** | | ***p-het*** | | |  | | | **HR (95% CI)** | | ***p-het*** | | |  | | | **HR (95% CI)** | | ***p-het*** | | |  | | | **HR (95% CI)** | | ***p-het*** | | |  | | | **HR (95% CI)** | | ***p-het*** | | |  | | | **HR (95% CI)** | | ***p-het*** | | | |  |
| **All-cause mortality** | | | | | | | | | | | | | | | | | | | | | | | | | | | | | | | | | | | | | | | | | | | | | | | | | | | | | | |  |  |
| Low-risk drinkers | . | 19368 | . | | 1277 | | . | | . | | 1.00 (0.94-1.06) | | *.* | | | . | | | 1.00 (0.94-1.06) | | *.* | | | | . | | 1.00 (0.94-1.06) | | *.* | | | | . | | 1.00 (0.94-1.06) | | *.* | | | | . | | 1.00 (0.94-1.06) | | *.* | | | | . | | 1.00 (0.94-1.06) | | *.* | | | |
| High-risk drinkers | . | 21262 | . | | 1610 | | . | | . | | 1.33 (1.27-1.40) | | *.* | | | . | | | 1.31 (1.25-1.38) | | *.* | | | | . | | 1.28 (1.22-1.35) | | *.* | | | | . | | 1.29 (1.22-1.35) | | *.* | | | | . | | 1.28 (1.22-1.34) | | *.* | | | | . | | 1.28 (1.22-1.34) | | *.* | | | |
| 1 problem drinking indicator | . | 9791 | . | | 1056 | | . | | . | | 1.47 (1.38-1.56) | | *.* | | | . | | | 1.42 (1.34-1.51) | | *.* | | | | . | | 1.38 (1.30-1.47) | | *.* | | | | . | | 1.37 (1.29-1.46) | | *.* | | | | . | | 1.37 (1.29-1.46) | | *.* | | | | . | | 1.37 (1.28-1.46) | | *.* | | | |
| 2+ problem drinking indicators | . | 2457 | . | | 345 | | . | | . | | 2.10 (1.88-2.33) | | *<.0001* | | | . | | | 2.01 (1.80-2.24) | | *<.0001* | | | | . | | 1.93 (1.73-2.15) | | *<.0001* | | | | . | | 1.92 (1.72-2.13) | | *<.0001* | | | | . | | 1.91 (1.71-2.12) | | *<.0001* | | | | . | | 1.91 (1.71-2.12) | | *<.0001* | | | |
| **All hospitalisations** | | | | | | | | | | | | | | | | | | | | | | | | | | | | | | | | | | | | | | | | | | | | | | | | | | | | | | |  |  |
| Low-risk drinkers | . | 10664 | . | | 9981 | | . | | . | | 1.00 (0.98-1.02) | | *.* | | | . | | | 1.00 (0.98-1.02) | | *.* | | | | . | | 1.00 (0.98-1.02) | | *.* | | | | . | | 1.00 (0.98-1.02) | | *.* | | | | . | | 1.00 (0.98-1.02) | | *.* | | | | . | | 1.00 (0.98-1.02) | | *.* | | | |
| High-risk drinkers | . | 11566 | . | | 11306 | | . | | . | | 1.11 (1.09-1.13) | | *.* | | | . | | | 1.11 (1.09-1.13) | | *.* | | | | . | | 1.10 (1.08-1.12) | | *.* | | | | . | | 1.10 (1.08-1.12) | | *.* | | | | . | | 1.09 (1.07-1.11) | | *.* | | | | . | | 1.09 (1.07-1.11) | | *.* | | | |
| 1 problem drinking indicator | . | 4494 | . | | 6353 | | . | | . | | 1.09 (1.06-1.12) | | *.* | | | . | | | 1.09 (1.06-1.12) | | *.* | | | | . | | 1.08 (1.05-1.11) | | *.* | | | | . | | 1.08 (1.05-1.11) | | *.* | | | | . | | 1.07 (1.04-1.10) | | *.* | | | | . | | 1.07 (1.04-1.10) | | *.* | | | |
| 2+ problem drinking indicators | . | 1138 | . | | 1664 | | . | | . | | 1.17 (1.12-1.23) | | *<.0001* | | | . | | | 1.17 (1.12-1.23) | | *<.0001* | | | | . | | 1.16 (1.11-1.22) | | *<.0001* | | | | . | | 1.16 (1.11-1.22) | | *<.0001* | | | | . | | 1.15 (1.10-1.21) | | *<.0001* | | | | . | | 1.15 (1.10-1.21) | | *<.0001* | | | |
| **External causes (ICD-10:V01-Y98)^a^** | | | | | | | | | | | | | | | | | | | | | | | | | | | | | | | | | | | | | | | | | | | | | | | | | | | | | | |  |  |
| Low-risk drinkers | . | 20530 | . | | 115 | | . | | . | | 1.00 (0.83-1.21) | | *.* | | | . | | | 1.00 (0.83-1.21) | | *.* | | | | . | | 1.00 (0.83-1.21) | | *.* | | | | . | | 1.00 (0.83-1.21) | | *.* | | | | . | | 1.00 (0.83-1.21) | | *.* | | | | . | | 1.00 (0.83-1.21) | | *.* | | | |
| High-risk drinkers | . | 22708 | . | | 164 | | . | | . | | 1.19 (1.02-1.39) | | *.* | | | . | | | 1.17 (1.00-1.37) | | *.* | | | | . | | 1.16 (0.99-1.35) | | *.* | | | | . | | 1.16 (0.99-1.36) | | *.* | | | | . | | 1.16 (0.99-1.36) | | *.* | | | | . | | 1.16 (0.99-1.36) | | *.* | | | |
| 1 problem drinking indicator | . | 10740 | . | | 107 | | . | | . | | 1.29 (1.06-1.56) | | *.* | | | . | | | 1.23 (1.01-1.49) | | *.* | | | | . | | 1.21 (1.00-1.47) | | *.* | | | | . | | 1.21 (0.99-1.46) | | *.* | | | | . | | 1.21 (1.00-1.47) | | *.* | | | | . | | 1.21 (0.99-1.47) | | *.* | | | |
| 2+ problem drinking indicators | . | 2756 | . | | 46 | | . | | . | | 2.13 (1.59-2.86) | | *0.0003* | | | . | | | 2.02 (1.51-2.71) | | *0.0012* | | | | . | | 1.98 (1.47-2.65) | | *0.0020* | | | | . | | 1.97 (1.47-2.64) | | *0.0022* | | | | . | | 1.98 (1.47-2.65) | | *0.0020* | | | | . | | 1.97 (1.47-2.64) | | *0.0022* | | | |
| Intentional self-harm (ICD-10: X60-X84)^a^ | | | | | | | | | | | | | | | | | | | | | | | | | | | | | | | | | | | | | | | | | | | | | | | | | | | | | | |  |  |
| Low-risk drinkers | . | 20636 | . | | 9 | | . | | . | | 1.00 (0.51-1.97) | | *.* | | | . | | | 1.00 (0.51-1.97) | | *.* | | | | . | | 1.00 (0.51-1.98) | | *.* | | | | . | | 1.00 (0.50-1.99) | | *.* | | | | . | | 1.00 (0.50-1.99) | | *.* | | | | . | | 1.00 (0.49-2.03) | | *.* | | | |
| High-risk drinkers | . | 22856 | . | | 16 | | . | | . | | 1.53 (0.93-2.50) | | *.* | | | . | | | 1.49 (0.90-2.44) | | *.* | | | | . | | 1.52 (0.92-2.49) | | *.* | | | | . | | 1.56 (0.95-2.56) | | *.* | | | | . | | 1.52 (0.92-2.49) | | *.* | | | | . | | 1.60 (0.97-2.64) | | *.* | | | |
| 1 problem drinking indicator | . | 10833 | . | | 14 | | . | | . | | 2.01 (1.18-3.43) | | *.* | | | . | | | 1.83 (1.07-3.13) | | *.* | | | | . | | 1.88 (1.10-3.20) | | *.* | | | | . | | 1.90 (1.12-3.25) | | *.* | | | | . | | 1.90 (1.12-3.25) | | *.* | | | | . | | 2.04 (1.19-3.50) | | *.* | | | |
| 2+ problem drinking indicators | . | 2797 | . | | 5 | | . | | . | | 2.87 (1.19-6.96) | | *0.2372* | | | . | | | 2.47 (1.02-5.98) | | *0.3831* | | | | . | | 2.56 (1.05-6.22) | | *0.3502* | | | | . | | 2.57 (1.06-6.24) | | *0.3471* | | | | . | | 2.60 (1.07-6.32) | | *0.3333* | | | | . | | 2.77 (1.14-6.78) | | *0.2804* | | | |
| Transport accident (ICD-10: V01-V99)^a^ | | | | | | | | | | | | | | | | | | | | | | | | | | | | | | | | | | | | | | | | | | | | | | | | | | | | | | |  |  |
| Low-risk drinkers | . | 20590 | . | | 55 | | . | | . | | 1.00 (0.76-1.31) | | *.* | | | . | | | 1.00 (0.76-1.32) | | *.* | | | | . | | 1.00 (0.76-1.32) | | *.* | | | | . | | 1.00 (0.76-1.32) | | *.* | | | | . | | 1.00 (0.76-1.32) | | *.* | | | | . | | 1.00 (0.76-1.32) | | *.* | | | |
| High-risk drinkers | . | 22790 | . | | 82 | | . | | . | | 1.16 (0.93-1.44) | | *.* | | | . | | | 1.14 (0.91-1.42) | | *.* | | | | . | | 1.13 (0.91-1.41) | | *.* | | | | . | | 1.13 (0.91-1.41) | | *.* | | | | . | | 1.14 (0.91-1.42) | | *.* | | | | . | | 1.14 (0.91-1.42) | | *.* | | | |
| 1 problem drinking indicator | . | 10802 | . | | 45 | | . | | . | | 1.07 (0.79-1.44) | | *.* | | | . | | | 1.03 (0.76-1.39) | | *.* | | | | . | | 1.02 (0.76-1.38) | | *.* | | | | . | | 1.02 (0.76-1.38) | | *.* | | | | . | | 1.03 (0.76-1.39) | | *.* | | | | . | | 1.03 (0.76-1.39) | | *.* | | | |
| 2+ problem drinking indicators | . | 2783 | . | | 19 | | . | | . | | 1.67 (1.06-2.63) | | *0.2826* | | | . | | | 1.60 (1.02-2.53) | | *0.3371* | | | | . | | 1.59 (1.01-2.50) | | *0.3553* | | | | . | | 1.59 (1.01-2.51) | | *0.3491* | | | | . | | 1.61 (1.02-2.54) | | *0.3370* | | | | . | | 1.61 (1.02-2.54) | | *0.3370* | | | |

HR, hazard ratio; CI, confidence interval; BMI, body mass index; ICD-10, international classification of diseases version 10; HED, heavy episodic drinking.

Participants with prior coronary heart disease, stroke, transient ischaemic attack, diabetes, cancer, tuberculosis, chronic hepatitis/cirrhosis, rheumatoid arthritis, peptic ulcer, emphysema/bronchitis, gallstone/gallbladder disease or kidney disease were excluded from the analysis.

Low-risk drinkers were current regular drinkers who drank <200g/week, with no HED in a typical drinking week or problem drinking indicator reported; high-risk drinkers were current regular drinkers who either drank at least 200g/week or engaged in HED in a typical drinking week, but with no problem drinking indicator reported; problem drinkers were current regular drinkers who reported at least one problem drinking indicator, and were further classified into “1 problem drinking indicator” and “2+ problem drinking indicators” according to the number of problem drinking indicators reported.

^a^ Deaths and hospitalisations combined

**Figure S9. Cross-sectional associations of problem drinking with wellbeing-related measures in men**

ORs were adjusted for age, region, education, income, marital status, prior chronic diseases, smoking and BMI. Each solid square represents an OR. 95% CIs are plotted using floating standard errors to allow for comparison between any two categories. The size of each box is inversely proportional to the ‘floated’ variance of the log OR in each group and the error bars indicate the group-specific 95% CI. OR, odds ratio; CI, confidence interval; BMI, body mass index; HED, heavy episodic drinking. Ex-regular drinkers (including reduced-intake drinkers) were men who had drunk alcohol ≥weekly previously but stopped so in the past year; abstainers were men who had not drunk alcohol in the past year and had not drunk ≥weekly previously; occasional drinkers were men who reported drinking alcohol less than weekly in the past year and had not drunk alcohol ≥weekly previously; low-risk drinkers were current regular drinkers (i.e., reported drinking ≥weekly in the past year) who drank <200g/week, with no HED in a typical drinking week or problem drinking indicator reported; high-risk drinkers were current regular drinkers who either drank at least 200g/week or engaged in HED in a typical drinking week, but with no problem drinking indicator reported; problem drinkers were current regular drinkers who reported at least one problem drinking indicator, and were further classified into “1 problem drinking indicator” and “2+ problem drinking indicators” according to the number of problem drinking indicators reported.

**Figure S10. Prospective associations of problem drinking with all-cause mortality, all hospitalisations and events due to all external causes in men without prior chronic diseases**

Models were stratified by age-at-risk and region, further adjusted for education, income, smoking, physical activity, fruit intake and BMI. Participants with prior coronary heart disease, stroke, transient ischaemic attack, diabetes, cancer, tuberculosis, chronic hepatitis/cirrhosis, rheumatoid arthritis, peptic ulcer, emphysema/bronchitis, gallstone/gallbladder disease or kidney disease were excluded from the analysis. Each solid square represents an HR. 95% CIs are plotted using floating standard errors to allow for comparison between any two categories. The size of each box is inversely proportional to the ‘floated’ variance of the log HR in each group and the error bars indicate the group-specific 95% CI. HR, hazard ratio; CI, confidence interval; BMI, body mass index; ICD-10, international classification of diseases version 10; HED, heavy episodic drinking. Ex-regular drinkers (including reduced-intake drinkers) were men who had drunk alcohol ≥weekly previously but stopped so in the past year; abstainers were men who had not drunk alcohol in the past year and had not drunk ≥weekly previously; occasional drinkers were men who reported drinking alcohol less than weekly in the past year and had not drunk alcohol ≥weekly previously. Low-risk drinkers were current regular drinkers (i.e., reported drinking ≥weekly in the past year) who drank <200g/week, with no HED in a typical drinking week or problem drinking indicator reported; high-risk drinkers were current regular drinkers who either drank at least 200g/week or engaged in HED in a typical drinking week, but with no problem drinking indicator reported; problem drinkers were current regular drinkers who reported at least one problem drinking indicator, and were further classified into “1 problem drinking indicator” and “2+ problem drinking indicators” according to the number of problem drinking indicators reported.

**Table S14. Baseline characteristics of men by alcohol drinking and problem drinking status**

|  | | | | | | | | | | | | | | **Current regular drinkers** | | | | | | | | | | | |
| --- | --- | --- | --- | --- | --- | --- | --- | --- | --- | --- | --- | --- | --- | --- | --- | --- | --- | --- | --- | --- | --- | --- | --- | --- | --- |
|  | | | | | | | | | | | | | | | | | **Non-problem drinkers** | | | |  | **Problem drinkers** | | | |
|  | | **All men** | |  | **Abstainers** | |  | **Ex-regular drinkers** | |  | **Occasional drinkers** | |  | **All current regular** | |  | **Low-risk** | | **High-risk** | |  | **1 indicator** | | **2+ indicators** | |
| Number of men (%) | . | 210259 |  | . | 42789 | (20.4) | . | 18294 | (8.7) | . | 79272 | (37.7) | . | 69904 | (33.2) | . | 25958 | (12.3) | 27254 | (13.0) | . | 13271 | (6.3) | 3421 | (1.6) |
| **Socio-demographic characteristics** | | | | | | | | | | | | | | | | | | | | | | | | | |
| Mean age, years (SD) | . | 52.4 | (10.9) | . | 55.8 | (11.1) | . | 56.4 | (10.3) | . | 50.7 | (10.8) | . | 51.0 | (10.2) | . | 51.7 | (10.7) | 50.2 | (9.8) | . | 51.8 | (10.1) | 50.9 | (9.8) |
| Age groups, years, % |  |  |  |  |  |  |  |  |  |  |  |  |  |  |  |  |  |  |  |  |  |  |  |  |  |
| <40 | . | 14.1 |  | . | 8.8 |  | . | 6.9 |  | . | 18.3 |  | . | 15.1 |  | . | 14.7 |  | 15.4 |  | . | 12.4 |  | 14.7 |  |
| 40-49 | . | 28.2 |  | . | 19.4 |  | . | 19.5 |  | . | 31.1 |  | . | 31.8 |  | . | 31.2 |  | 35.2 |  | . | 31.2 |  | 33.2 |  |
| 50-59 | . | 30.3 |  | . | 29.0 |  | . | 32.9 |  | . | 28.5 |  | . | 31.2 |  | . | 29.8 |  | 31.4 |  | . | 33.0 |  | 34.6 |  |
| 60-69 | . | 19.7 |  | . | 28.2 |  | . | 28.9 |  | . | 15.9 |  | . | 16.4 |  | . | 17.6 |  | 14.0 |  | . | 17.6 |  | 14.2 |  |
| 70+ | . | 7.8 |  | . | 14.7 |  | . | 11.7 |  | . | 6.0 |  | . | 5.5 |  | . | 6.8 |  | 4.0 |  | . | 5.7 |  | 3.2 |  |
| Urban area, % | . | 43.4 |  | . | 31.2 |  | . | 41.1 |  | . | 44.1 |  | . | 50.0 |  | . | 61.3 |  | 51.9 |  | . | 30.5 |  | 24.0 |  |
| Educational attainment >6y, % | . | 57.8 |  | . | 54.5 |  | . | 56.7 |  | . | 60.5 |  | . | 57.6 |  | . | 63.1 |  | 60.0 |  | . | 57.7 |  | 57.5 |  |
| Income >20000yuan/year, % | . | 45.6 |  | . | 42.0 |  | . | 44.9 |  | . | 46.7 |  | . | 46.8 |  | . | 52.7 |  | 52.8 |  | . | 47.5 |  | 46.8 |  |
| Married, % | . | 92.9 |  | . | 91.4 |  | . | 93.4 |  | . | 93.3 |  | . | 93.3 |  | . | 94.8 |  | 93.9 |  | . | 92.6 |  | 91.1 |  |
| **Lifestyle & physical measurements** | | | | | | | | | | | | | | | | | | | | | | | | | |
| Regular smoking, % | . | 61.1 |  | . | 52.3 |  | . | 60.4 |  | . | 56.9 |  | . | 71.7 |  | . | 64.9 |  | 74.1 |  | . | 76.2 |  | 79.0 |  |
| Mean physical activity, MET-h/d (SD) | . | 22.0 | (15.3) | . | 21.1 | (15.1) | . | 20.2 | (14.5) | . | 22.6 | (15.6) | . | 22.4 | (15.0) | . | 22.9 | (14.8) | 22.8 | (14.9) | . | 23.1 | (15.2) | 23.4 | (15.7) |
| Mean SBP, mmHg (SD) | . | 132.8 | (20.0) | . | 132.3 | (21.5) | . | 134.3 | (21.5) | . | 131.0 | (18.8) | . | 134.8 | (19.8) | . | 131.8 | (19.1) | 135.7 | (19.7) | . | 135.3 | (20.6) | 136.7 | (20.9) |
| Mean BMI, kg/m^2^ (SD) | . | 23.4 | (3.2) | . | 23.3 | (3.2) | . | 23.9 | (3.4) | . | 23.4 | (3.2) | . | 23.4 | (3.2) | . | 23.6 | (3.2) | 23.9 | (3.3) | . | 23.5 | (3.2) | 23.3 | (3.1) |
| **Medical history, %** | | | | | | | | | | | | | | | | | | | | | | | | | |
| CHD | . | 2.7 |  | . | 3.3 |  | . | 5.2 |  | . | 2.3 |  | . | 2.0 |  | . | 2.0 |  | 1.7 |  | . | 1.9 |  | 3.0 |  |
| Stroke or TIA | . | 2.3 |  | . | 3.6 |  | . | 6.0 |  | . | 1.6 |  | . | 1.3 |  | . | 1.3 |  | 1.2 |  | . | 1.4 |  | 2.4 |  |
| Cancers | . | 0.5 |  | . | 0.9 |  | . | 1.1 |  | . | 0.3 |  | . | 0.3 |  | . | 0.3 |  | 0.2 |  | . | 0.2 |  | 0.2 |  |
| Chronic hepatitis or cirrhosis | . | 1.7 |  | . | 2.7 |  | . | 3.8 |  | . | 1.6 |  | . | 1.2 |  | . | 1.2 |  | 1.1 |  | . | 1.4 |  | 1.4 |  |
| Diabetes | . | 2.9 |  | . | 3.8 |  | . | 6.1 |  | . | 2.6 |  | . | 1.9 |  | . | 2.1 |  | 2.0 |  | . | 1.8 |  | 2.5 |  |
| Psychiatric disorder | . | 0.3 |  | . | 0.6 |  | . | 0.3 |  | . | 0.2 |  | . | 0.2 |  | . | 0.1 |  | 0.1 |  | . | 0.2 |  | 0.4 |  |

SD, standard deviation; MET-h/d, metabolic equivalent of task per hour per day; SBP, systolic blood pressure; DBP, diastolic blood pressure; BMI, body mass index; CHD, coronary heart disease; TIA, transient ischaemic attack; HED, heavy episodic drinking.

Prevalences and means are adjusted for age group and region as appropriate by direct standardisation to the age and region structure of all men for drinking categories (abstainers, ex-regular drinkers, occasional drinkers, current regular drinkers), and of current regular drinkers for problem drinking status (low-risk, high-risk, 1 indicator, 2+ indicators).

Associations between drinking categories and baseline characteristic variables evaluated with a chi-square test for association: p<0.0001 across all variables

Abstainers were men who had not drunk alcohol in the past year and had not drunk ≥weekly previously; ex-regular drinkers were men who had drunk alcohol ≥weekly previously but stopped so in the past year; occasional drinkers were men who reported drinking alcohol less than weekly in the past year and had not drunk alcohol ≥weekly previously; low-risk drinkers were current regular drinkers (i.e., reported drinking ≥weekly in the past year) who drank <200g/week, with no HED in a typical drinking week or problem drinking indicator reported; high-risk drinkers were current regular drinkers who either drank at least 200g/week or engaged in HED in a typical drinking week, but with no problem drinking indicator reported; problem drinkers were current regular drinkers who reported at least one problem drinking indicator, and were further classified into “1 problem drinking indicator” and “2+ problem drinking indicators” according to the number of problem drinking indicators reported.

**Table S15. Comparison of problem drinking and AUD definitions in the China Kadoorie Biobank and commonly used screening tests and diagnostic criteria**

|  | **DSM-5** | **CKB** | **CAGE** | **AUDIT** |
| --- | --- | --- | --- | --- |
| **Definition** | **AUD (in the past year): -** Mild (2-3 items);   - Moderate (4-5 items); - Severe (6+ items). | **Problem drinking (in the past month) :** ≥1 problem drinking indicator(s) | **Problem drinking (lifetime):** ≥2 ”yes” responses | **Hazardous or harmful alcohol consumption:** score ≥8+ (men) or ≥7 (women);  **Alcohol dependence (in the past year):** score ≥20. |
| **Indicators related to amount** |  |  |  | - How often did you have a drink containing alcohol in the past year? |
|  |  |  |  | - How many drinks containing alcohol did you have on a typical day when you were drinking in the past year? |
|  |  |  |  | - How often did you have 6 or more drinks on one occasion in the past year? |
| **Indicators related to alcohol dependence and problems** |  | - Ever drinking in the morning | - Have you ever felt you needed a drink first thing in the morning (**E**ye-opener) to steady your nerves or to get rid of a hangover? | - How often during the last year have you needed a first drink in the morning to get yourself going after a heavy drinking session? |
|  | - Experienced craving — a strong need, or urge, to drink? |  |  |  |
|  | - Had times when you ended up drinking more, or longer than you intended? | - Unable to keep away from drinking |  | - How often during the last year have you found that you were not able to stop drinking once you had started? |
|  | - More than once wanted to cut down or stop drinking, or tried to, but couldn’t? |  | - Have you ever felt you needed to **C**ut down on your drinking? |  |
|  | - Spent a lot of time drinking? Or being sick or getting over the after effects? |  |  |  |
|  | - Had withdrawal symptoms e.g. trouble sleeping, shakiness, irritability, anxiety, depression, restlessness, nausea, or sweating? Or sensed things that were not there? | - Having shakes when stopping drinking |  |  |
|  | - Had to drink much more than you once did to get the effect you want? Or found that your usual number of drinks had much less effect than before? |  |  |  |
|  | - Continued to drink even though it was making you feel depressed or anxious or adding to another health problem? Or after having had a memory blackout? | - Negative emotions after drinking |  | - How often during the last year have you been unable to remember what happened the night before because you had been drinking? |
|  | - Given up or cut back on activities that were important or interesting to you, or gave you pleasure, in order to drink? |  |  |  |
|  | - Found that drinking - or being sick from drinking - often interfered with taking care of your home or family? Or caused job troubles? Or school problems? | - Unable to work or to do anything because of drinking |  | - How often during the last year have you failed to do what was normally expected from you because of drinking? |
|  |  |  | - Have you ever felt **G**uilty about drinking? | - How often during the last year have you had a feeling of guilt or remorse after drinking? |
|  | - More than once gotten into situations while or after drinking that increased your chances of getting hurt (e.g. driving, swimming, using machinery, walking in a dangerous area, or having unsafe sex)? |  |  | - Have you or someone else been injured as a result of your drinking? |
|  | - Continued to drink even though it was causing trouble with your family or friends? |  | - Have people **A**nnoyed you by criticizing your drinking? | - Has a relative or friend or doctor or other health care worker been concerned about your drinking or suggested you cut down? |

AUD: alcohol use disorder; AUDIT: alcohol use disorder identification test; DSM-5: diagnostic and statistical manual of mental disorders-5; CAGE: cut down, annoyed, guilty, eye-opener.

AUDIT scoring system: each question is based on a 0-4 point scale (0=”Never”, 1=”Less than monthly”, 2=”Monthly”, 3=”Weekly”, 4=”Daily/almost daily”)

**Reference**

1. Mitchell J, Rennie KL, Day NE, Wareham NJ, Jakes RW, Hennings S: Validity and repeatability of the EPIC-Norfolk Physical Activity Questionnaire. *International Journal of Epidemiology* 2002, 31(1):168-174.

2. Matthews CE, Shu XO, Yang G, Jin F, Ainsworth BE, Liu D, Gao YT, Zheng W: Reproducibility and validity of the Shanghai Women's Health Study physical activity questionnaire. *Am J Epidemiol* 2003, 158(11):1114-1122.
